# An electrically actuated molecular toggle switch

**DOI:** 10.1038/ncomms14672

**Published:** 2017-03-09

**Authors:** Lukas Gerhard, Kevin Edelmann, Jan Homberg, Michal Valášek, Safa G. Bahoosh, Maya Lukas, Fabian Pauly, Marcel Mayor, Wulf Wulfhekel

**Affiliations:** 1 Institut für Nanotechnologie, Karlsruhe Institute of Technology, 76344 Eggenstein-Leopoldshafen, Germany; 2 Physikalisches Institut, Karlsruhe Institute of Technology, 76131 Karlsruhe, Germany; 3 Department of Physics, University of Konstanz, 78457 Konstanz, Germany; 4 Department of Chemistry, University of Basel, St Johanns-Ring 19, 4056 Basel, Switzerland; 5 Lehn Institute of Functional Materials (LIFM), Sun Yat-Sen University, Guangzhou 510275, China

## Abstract

Molecular electronics is considered a promising approach for future nanoelectronic devices. In order that molecular junctions can be used as electrical switches or even memory devices, they need to be actuated between two distinct conductance states in a controlled and reproducible manner by external stimuli. Here we present a tripodal platform with a cantilever arm and a nitrile group at its end that is lifted from the surface. The formation of a coordinative bond between the nitrile nitrogen and the gold tip of a scanning tunnelling microscope can be controlled by both electrical and mechanical means, and leads to a hysteretic switching of the conductance of the junction by more than two orders of magnitude. This toggle switch can be actuated with high reproducibility so that the forces involved in the mechanical deformation of the molecular cantilever can be determined precisely with scanning tunnelling microscopy.

## Abstract (web summary)

Robust molecular junctions demand highly reproducible switching between two or more well-defined conductance states upon control. Here, Gerhard *et al*. show the utility of elastic deformation of tripodal spirobifluorene derivatives in the junction of a scanning tunnelling microscope to achieve this goal.

The formation of a molecular junction and thus ultimately the performance of a single-molecule device is determined by quantum mechanics. Thus, junctions of chemically identical molecules should behave identically allowing for a reliable operation, which is one of the fundamental advantages of molecular electronics^1^,^2^. In the past, break-junction experiments largely contributed to the progress of understanding electron transport in single-molecule junctions^3^,^4^,^5^. In these measurements, however, the molecular adsorption and the arrangement of the atoms in the metallic electrodes are *a priori* unknown and often many different nearly degenerate junction geometries exist. This in turn leads to large variations of the electronic properties of the individual junctions and requires a statistical analysis of the data^6^. Furthermore, break-junction techniques inherently lack the possibility of lateral resolution, which neither allows to directly identify the molecule of interest at the electrode surface nor to deliberately explore variations in bond configurations. Reproducible performance and well-defined characteristics of the molecular junction, however, are prerequisites for both the comprehension of molecular devices and their implementation into future electronic devices^7^,^8^. Recently, scanning tunnelling microscope (STM) and atomic force microscope (AFM) set-ups^9^ emerged as a powerful alternative to break-junction methods. In this approach, the adsorption geometry of the molecules can be determined before contacting a molecule with the tip at specific positions^10^,^11^,^12^,^13^,^14^. Forces in the range of piconewtons that result from the onset of bond formation can be derived from the frequency shift of the cantilever of an AFM oscillating by some tens to some hundreds of picometres^12^,^14^,^15^.

With a view to future applications, it is necessary to control the properties of single-molecule junctions by external means^16^ such as mechanical forces^17^, electric fields^18^,^19^,^20^, currents^21^,^22^ or light^23^,^24^. In this respect, it is helpful to design the molecule specifically such that it binds to the substrate electrode in a well-defined geometry and to lift the functional group of the molecule from the metallic substrate. This can be achieved by three-dimensional molecular carrier platforms that allow for a well-defined arrangement of molecular subunits with respect to the substrate. Tremendous progress in the synthesis of such molecules has been made recently^25^,^26^,^27^,^28^,^29^.

In this work, we present low-temperature STM measurements and density functional theory (DFT) calculations on tripodal spirobifluorene derivatives (spiro), which are designed to firmly bind to a gold surface via three sulfur atoms at the feet^27^. They carry, as functional subunit, a freely suspended nitrile group that is well separated from the metallic substrate, freely accessible by the STM tip and mounted on a rigid subunit. In the following, we show that the high reproducibility of the process of contact formation of the nitrile group to a gold electrode allows us to build a fully controllable electromechanical molecular toggle switch and to deduce the forces that are needed to stretch the molecular junction.

## Results

### Deposition of spiro A and spiro B molecules

**Figure 1:**
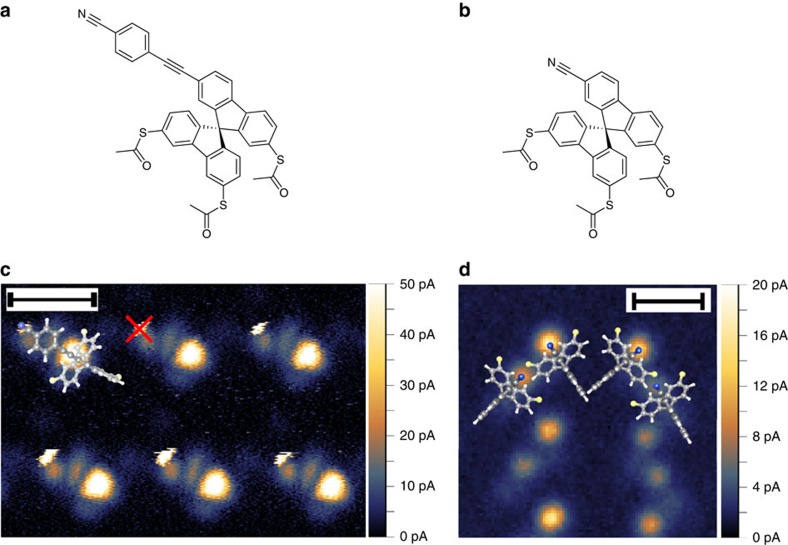
Adsorption geometry of the tripodal molecules. (**a**,**b**) Chemical structure of spiro A and spiro B as synthesized. (**c**,**d**) STM image of ordered layers of spiro A and spiro B recorded in constant height mode at a bias voltage of 1.9 V and 700 mV with the corresponding molecular models superimposed to scale. A red cross indicates a typical position for contact formation. The length of the scale bars is 1 nm.

The molecules, entitled spiro A and spiro B, are presented in Fig. 1a,b, and their design comprises a firmly immobilized platform, exposing a rigidly mounted freely suspended cantilever. They were deposited onto clean Au(111) surfaces by a spray deposition method described previously^27^, followed by mild annealing in ultra-high vacuum (UHV), which promotes the deprotection of the sulfur anchor groups and the proper binding to the gold substrate. Both molecules arrange in ordered islands with a well-defined adsorption geometry (Fig. 1c,d). While the synthesis of spiro A is already reported^27^, the synthesis of spiro B is provided in the Methods section. Details of the adsorption geometry are presented in Supplementary Fig. 14 and Supplementary Note 9. Simulated STM images are discussed in Supplementary Figs 12 and 13 and Supplementary Note 8.

### Mechanical opening and closing of the molecular junctions

**Figure 2:**
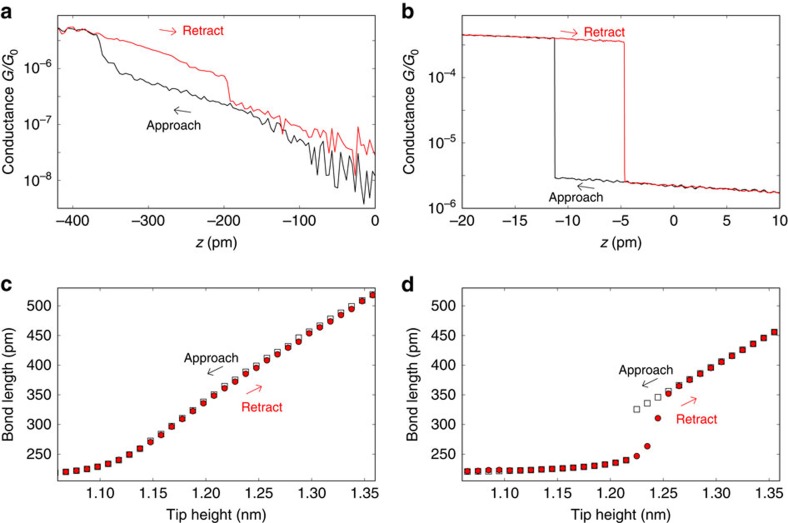
Contact formation by approaching the tip towards the sample. (**a**) *I–z* curve recorded above the head group of spiro A at a bias of 300 mV. *z* values are given with respect to the starting position (*z*=0), and the sweep direction is indicated by arrows. (**b**) *I–z* curve recorded above the head group of spiro B at a bias of 250 mV. (**c**,**d**) DFT calculations of the opening and closing of molecular junctions containing spiro B as a result of the tip movement with **c**, all S atoms fixed in their positions, and **d**, all S atoms free to move in the plane *z*=0, providing an enhanced mechanical flexibility. Plotted is the distance between the nitrogen atom of the molecular head group and the gold tip atom as a function of the tip height above the surface at *z*=0.

To study the distance-controlled contact formation to the nitrile group, we recorded the current while the STM tip was approached towards the molecule (black curves in Fig. 2a,b) and was retracted back to its initial position (red curves in Fig. 2a,b)^30^,^31^. At a certain critical distance *z*_ON_, the molecular contact closes and the measured conductance jumps to a higher value (−380 and −11 pm in Fig. 2a,b). Upon retraction, at a larger distance *z*_OFF_ between tip and sample (−180 and −4 pm in Fig. 2a,b), the contact reopens and the conductance jumps back to a lower value. The experimentally observed hysteretic behaviour results from the short-range binding forces between the tip and the nitrile group of the molecule combined with elastic deformations of the molecular bridge, which comprises also surface atoms of the tip and substrate. We manage to extract the forces involved in this purely elastic deformation of the molecular junction as described at the end of the manuscript.

To further investigate the bistable behaviour found in the molecular junctions, we performed DFT calculations of junction opening and closing processes (see the Methods section for details on the applied DFT procedures). In particular, we investigated whether the molecule alone is responsible for the hysteresis in conductance–displacement curves, as measured in Fig. 2b, or to what extent the electrodes and molecule–electrode interfaces need to be involved in the ON-to-OFF-state switching. To perform efficient calculations, we assumed that a single Au atom represents the tip. The S atoms of spiro B were assumed to be either completely fixed in the plane *z*=0 or free to move to describe the role of the substrate. The Au atom was then placed ∼2 Å directly above the N atom of a fully relaxed molecule. Subsequently, it was brought out of and into contact by moving it along the *z* axis away in steps of 0.1 Å until the contact broke and the molecule did not feel the tip. Afterwards, the procedure was reversed and the tip approached the molecule until the contact formed again and the Au atom reached its starting position. We computed the change in the Au–N bond length as a function of the height of the Au atom above the *z*=0 plane, as displayed in Fig. 2c,d. In the case that all S atoms are fixed completely, we find practically no hysteresis (Fig. 2c). But if the S atoms are allowed to move in the *x*–*y* plane, the legs gain more flexibility and a pronounced hysteresis is observed (Fig. 2d). This shows that also the assumed anchoring of the molecule to the substrate plays a crucial role for the junction bistability, or in other words the mechanical properties of the molecule–electrode interfaces as well as those of the electrodes. Generally speaking, the energy surface at those tip distances, where the hysteresis is observed, must exhibit a double-well potential since both ON and OFF states are stable, of rather similar energy but separated by an energy barrier, thus allowing the state of the system to depend on process history. This means that the elastic energy in the molecular junction must be close to the energy of the Au–N bond between the tip and the molecular nitrile group.

**Figure 3:**
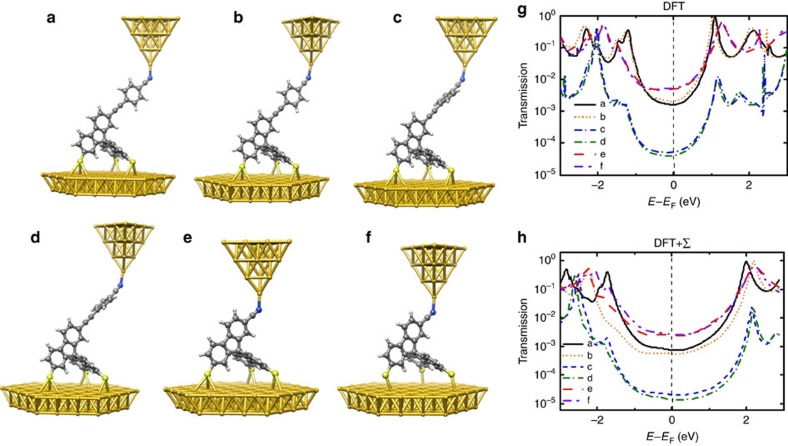
Transport through tripodal molecules on Au(111) in different configurations. (**a**–**d**) Spiro A. (**e**,**f**) Spiro B. (**g**) Computed transmission curves in DFT and (**h**) DFT+Σ frameworks are shown for configurations **a**–**f**.

At first sight, it can be seen that the conductance of spiro A is markedly lower than that of spiro B (5 × 10^−6^*G*_0_ in Fig. 2a compared to 4 × 10^−4^*G*_0_ in Fig. 2b, where the conductance is given in units of *G*_0_=2*e*^2^/*h*). To better understand this difference, we have computed the conductance of spiro A and spiro B with the DFT^32^ and DFT+Σ methods^33^,^34^,^35^. As described further in the Methods section, the conductance can be obtained from the energy-dependent transmission function *τ*(*E*) via equation (4), if phase-coherent transport is assumed. This reduces to *G*≈*G*_0_*τ*(*E*_F_) at low temperatures with the Fermi energy *E*_F_, and the conductance can thus be estimated from energy-dependent transmission curves. They are shown for different junction geometries of spiro A and B in Fig. 3, and values of the conductance (at vanishing temperature) are listed in Table 1. We observe that the short molecule spiro B shows a larger conductance than spiro A, as expected for off-resonant transport. A rotation of the assumed atomically sharp tip has basically no influence on transport, but a rotation of the phenyl ring at the head, that may be induced by tip–molecule interactions, leads to a strong suppression of the conductance of spiro A by more than one order of magnitude for perpendicular ring orientation. We observe that the conductance values determined from DFT are consistently a factor of around 2–4 larger than those of DFT+Σ. This results from an opening of the highest occupied molecular orbital–lowest unoccupied molecular orbital gap that is clearly visible for *τ*(*E*) in Fig. 3g,h. DFT+Σ results have been reported to show a good agreement with experimental conductance values^33^,^34^,^35^, since the DFT+Σ method is constructed to yield improved quasiparticle energies for the molecule in the metallic junction. For this reason, we will compare below only the DFT+Σ results to the experiment. Further details of the DFT+Σ calculations can be found in the Methods section, and Supplementary Note 7 and Supplementary Fig. 11.

To summarize Table 1 and compare to the experiment, values found for spiro B for different tip configurations are centred ∼2.65 × 10^−3^*G*_0_, that is, a factor of 7 above the experimental results. As the terminal phenyl ring of spiro A is freely revolving around the ethynyl connection, we also considered the twisted arrangement of the phenyl ring. In its untwisted configuration, the conductance is ∼6.5 × 10^−4^*G*_0_, which is larger than the experimental value by a factor of 130. A calculation for a twist angle of 90° leads to a value of ∼1.75 × 10^−5^*G*_0_ which is, similar to the case of spiro B, within an order of magnitude from the measurements. In full agreement with the simulated STM images presented in the Supplementary Figs 12 and 13, this indicates that the terminal phenyl ring twists in the presence of the tip.

**Figure 4:**
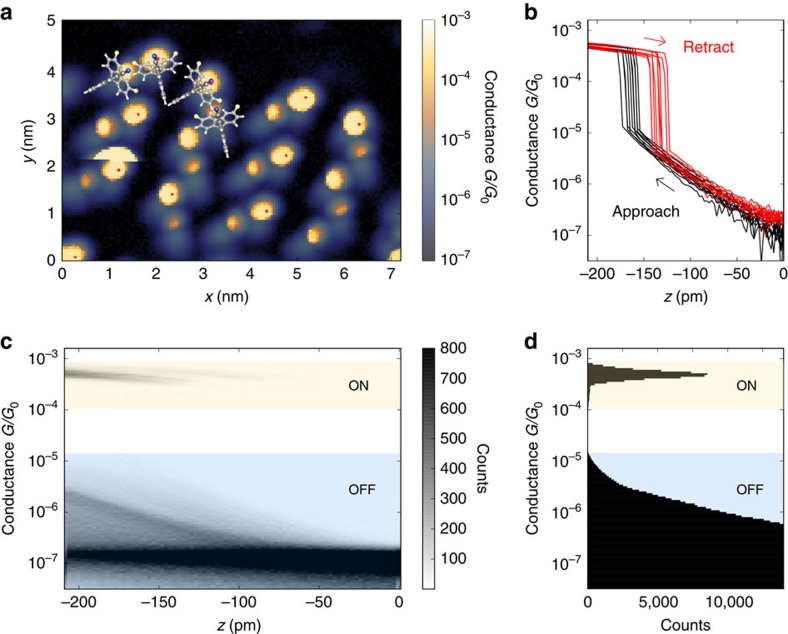
Reproducibility of contact formation. (**a**) Conductance measurements with *I–z* curves recorded at every pixel above spiro B molecules at a bias of 70 mV. The colour code shows the conductance at the closest distance (*z*=−210 pm) with molecular structure superimposed to scale. (**b**) Ten *I–z* curves recorded on equivalent positions of 10 different molecules marked by red dots in **a**. (**c**) Two-dimensional histogram of all 14,000 approach and retraction curves. (**d**) One-dimensional histogram showing the clearly separated conductance values of the ON and the OFF state including all *I–z* curves.

Typically, such contacting measurements involve plastic deformations of the electrodes or displacements of the molecule, which leads to a widespread range of conductance values of the junction. Robust and reproducible conductance switching, however, is essential with regard to possible applications and requires suitable anchoring groups as is the case for the molecular tripods discussed in this work. On one side, the spiro molecules are firmly attached to the substrate via three sulfur bonds, and on the other side, a nitrile group forms a coordinative bond to the gold tip that can be reproducibly opened and closed. Several thousand conductance curves were recorded on an array of spiro B molecules and all molecular junctions formed resulted in basically the same value of conductance as can be seen in the conductance map presented in Fig. 4a, where all molecular head groups show up in identical colour. *I–z* curves recorded on equivalent positions of different molecules show identical switching behaviour (Fig. 4b). As can be seen in the corresponding conductance histograms (see Fig. 4c,d, including all 14,000 *I–z* curves on top of the molecular head group, near to it, on the side of the molecule and above the gold surface), the ON–OFF ratio of the switch amounts to about two orders of magnitude with no overlap of the corresponding conductance peaks. The high reproducibility of the nitrile–gold contact in the STM experiment goes well beyond break-junction experiments of molecules with similar anchor groups or similar core structures^36^,^37^. It allows to unambiguously attribute the remaining variation of the conductance of the ON state to the difference in atomic-scale contact geometries for different tip positions (see lateral mapping of the hysteresis described in detail in Supplementary Fig. 15 and Supplementary Note 10).

### Electrical opening and closing of the molecular junctions

**Figure 5:**
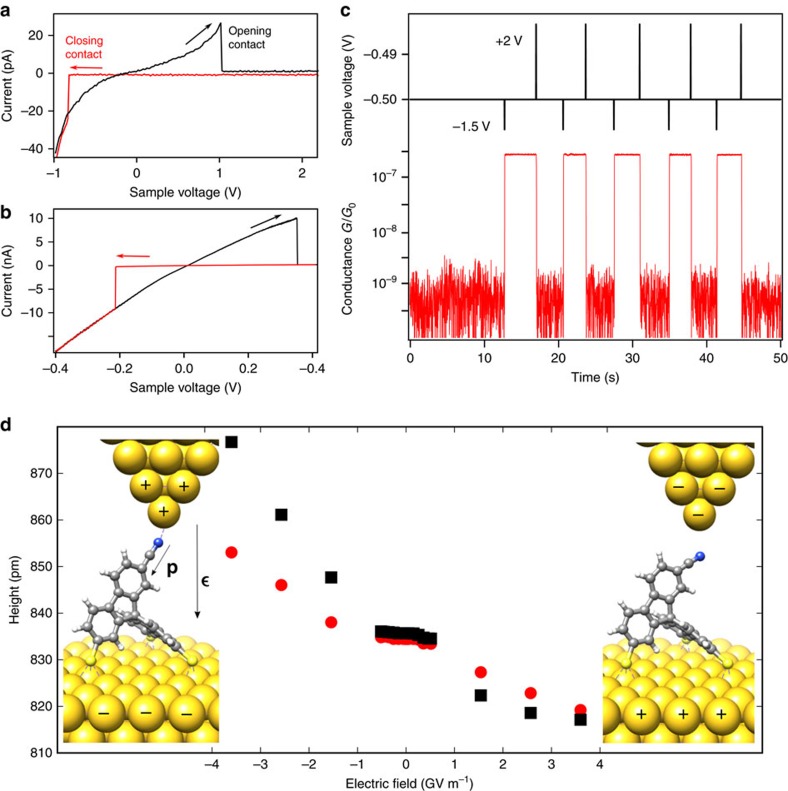
Contact formation by application of electric fields. (**a**,**b**) *I–V* curves at the position of the head group of spiro A (**a**) and spiro B (**b**). The arrows indicate the direction of the voltage ramp. (**c**) Time traces of applied voltage and resulting current during bistable switching between the two states of spiro A (open and closed junction) by voltage pulses of 130 μs and +2 V/−1.5 V. Recorded with an integration time of 20 ms. (**d**) Simulated response of spiro B to an external electric field. The *z* component of the N atom above the plane of the three S atoms at *z*=0 is shown as a function of the applied field. Red circles represent a spiro B molecule with all S atoms fixed, while the black squares are for the case, where S atoms can move freely in the *x*–*y* plane at *z*=0. Definition of the dipole moment **p** and the electric field ***ɛ*** as indicated in the molecular models.

**Figure 6:**
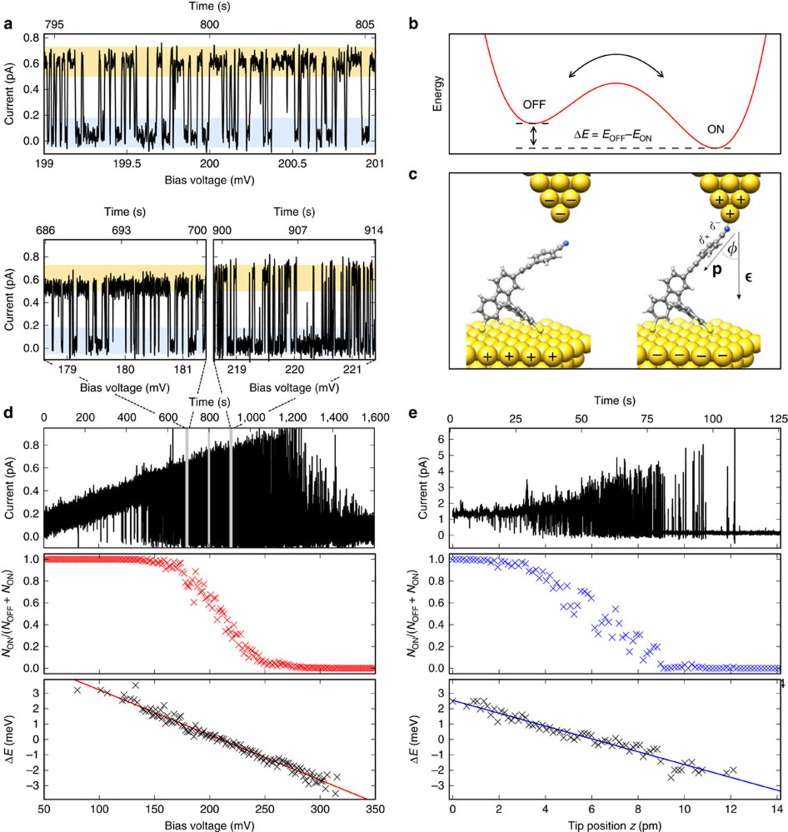
Energy differences between the open and closed molecular contact. (**a**) Statistical conductance switching of spiro A at a bias voltage of 200 mV. (**b**) Model of a double-well potential for ON and OFF states of the molecular head group. (**c**) Molecular model for the two states including definitions of the dipole moment **p**, the electric field ***ɛ*** and the angle *Φ*. (**d**) Statistical switching as a function of applied bias voltage and time. In addition to the enlarged view, shown in **a**, that corresponds to the grey line in the middle, two more voltage intervals marked in grey are shown in enlarged views (180 and 220 mV). The corresponding population of the ON state *N*_ON_/(*N*_OFF_+*N*_ON_) is indicated by red crosses, the resulting energy difference between ON and OFF states derived from Boltzmann statistics is shown by black crosses and the red line is a linear fit to the data. (**e**) Statistic switching recorded at 600 mV as a function of time and distance between tip and sample, corresponding population of the ON state (blue crosses) and resulting energy difference between ON and OFF states (black crosses, linear fit in blue).

The opening and closing of the molecular junction can also be controlled by the applied bias. As shown in Fig. 5a,b, *I*–*V* curves recorded above the head group exhibit a hysteretic switching between high- and low-conductance curves. The critical voltages that are necessary to open and close the molecular junction strongly depend on the lateral and the vertical position of the tip and range from −1 to 0.5 V and −0.7 to 2 V for closing and opening the junction, respectively (for details, see Supplementary Figs 16 and 17 and Supplementary Note 11). This behaviour excludes switching mechanisms that are related to a particular energy of the electrons such as inelastic excitations or tunnelling to specific orbitals^38^,^39^. However, it can be explained by the electric field that acts on the dipole moment of the molecular head group as follows: at positive bias voltages applied to the sample, the electric field points from the sample towards the tip and exerts a torque on the molecular dipole that in turn pushes the molecular head group away from the tip (Figs 5d and 6c). When the molecule is stretched, the elastic energy that counteracts the binding energy in the closed state increases and the contribution of the electrostatic energy becomes crucial. As soon as open and closed configurations of the junction are similar in energy, the electric field can break the nitrile–gold bond and opens the contact between tip and molecule, which leads to a sudden drop in the conductance at a voltage of 1 V for spiro A in Fig. 5a (0.35 V for spiro B in Fig. 5b). Similarly, at negative bias voltages, the electric field points towards the sample and pulls the head group towards the tip. This closes the tip–molecule contact, and the lone pair of the terminal nitrile nitrogen forms a coordinative bond to the gold tip, which is reflected in a sudden increase in the conductance at −0.8 V for spiro A in Fig. 5a (−0.2 V for spiro B in Fig. 5b). The effect is due to a purely elastic deformation in contrast to the electric-field-induced isomerization in azobenzene molecules^18^ or the recently described oxidation–reduction processes of organometallic compounds^20^.

A series of controlled switching events induced by voltage pulses of +2 V/−1.5 V with a read-out voltage of −0.5 V is shown in Fig. 5c, recorded on spiro A. The tip was placed directly over the nitrile group such that the read-out voltage lies within the voltage hysteresis, which allows bistability with an ON–OFF ratio of the conductance switch of more than two orders of magnitude. Note that every voltage pulse of 130 μs leads to switching and the final state of the junction solely depends on the polarity of the pulse, which makes this junction an ideal memristor. The short switching times indicate an efficient electric-field-driven switching mechanism opposed to that of current-induced switching in spin-crossover memristors^22^. Because the underlying switching mechanism does not involve plastic deformation, similar to the mechanical control presented above, the electric field allows to switch between two well-defined and distinct conductance values.

To theoretically examine these results, we first evaluated the dipole moments of benzonitrile as well as of the gas phase relaxations of spiro A and B molecules presented in Supplementary Fig. 8 and Supplementary Note 4. The absolute values of the dipole moments are shown in Table 2, and the angles *Φ*_dip_ between the surface normal (*z* direction) and the dipole moment in Supplementary Table 1. We find that absolute values of the dipole moment of the spiro molecules are similar to those of benzonitrile^40^. The angle between surface normal and the molecular head group is close to the angle between the surface normal and the dipole moment (for details, see Supplementary Table 1), indicating that the dipole moment of the spiro molecules, indeed, arises mainly from the benzonitrile motive at its head.

With regard to the influence of electric fields, we performed geometry optimizations of the spiro B molecule in a uniform, static electric field within DFT. We assume that the three sulfur atoms of the molecule are located in the *x*–*y* plane at *z*=0. In Fig. 5d, we show the *z* height of the terminal N atom as a function of the electric field applied along the *z* direction, similar to the geometry shown on the sides in Fig. 5d. Two cases of constrained relaxation are distinguished. In one case, the three S atoms in the legs are completely fixed, and in the other they are just constrained to *z*=0 and can move freely in the *x*–*y* plane otherwise. While the changes in height are somewhat smaller for the molecule with all S atoms fixed, as expected due to the less flexible geometry, the qualitative features are the same for both: the height of the N atom is reduced for positive fields and increased for negative ones. In perfect consistency with the experimental findings, this can be interpreted in terms of the alignment of the dipole of the nitrile group in the electric field. We also observe that the displacements of the N as compared to its equilibrium position at zero electric field are slightly larger at large negative electric field strength than those at a comparable but positive one. This asymmetry means that it is somewhat easier to stretch the molecule than to compress it.

### Deducing forces from statistical conductance switching

With the tip centrally placed above the molecular head group, formation and breaking of the nitrile–gold bond leads to switching between two metastable states. However, the short-range bond energy and thus the energy barrier between the two states are drastically reduced, if the tip is placed laterally in front of the nitrile group (Supplementary Fig. 10; Supplementary Note 6). Then, the application of electric fields allows us to fine-tune the energy balance between the two states of the molecular junction such that statistical switching between the two states is observed. Figure 6a shows an example of a current trace, where the junction statistically opens and closes. In addition to the arguments given in the description of the voltage-induced contact formation experiments shown in Fig. 5, the very low currents (<0.1 pA in the OFF state) strongly suggest thermally activated switching and exclude inelastic excitations as the origin of the switching. Furthermore, these low-current densities are unlikely to lead to a considerable increase of the temperature of the molecular junction. The thermal population of the two states in the double-well potential (Fig. 6b) is given by Boltzmann statistics:


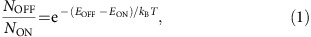


with *k*_B_ the Boltzmann constant, *E*_OFF_ and *E*_ON_ the energy of the OFF state, and the ON state, respectively. At a known temperature *T*, this relation allows us to calculate the difference between the energy levels *E*_OFF_−*E*_ON_ from the population of the two states. The energy landscape of our system can be described by a double-well potential formed by a sum of three contributions:


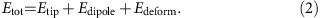


*E*_tip_ is the effective potential for the interaction of the molecular head group with the tip. The energy of an electric dipole **p** in an electric field ***ɛ***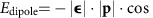 is given by 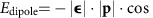
*Φ* with 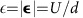, where *U* is the bias voltage, *d* the distance between tip and sample, and *Φ* the angle between the dipole and the electric field (Fig. 6c). Please note that we follow the convention of the dipole moment given by the International Union of Pure and Applied Chemistry (IUPAC) pointing from the negative to the positive charge, while in chemistry often the opposite definition of the direction of a molecular dipole is used. The energy for the elastic deformation of the molecular junction can be approximated to leading order by *E*_deform_=0.5*k*(*z*−*z*_0_)^2^, with *k* the effective stiffness and *z*_0_ the height of the relaxed molecule.

Following this model, we first analyse the energy difference as a function of the applied bias voltage, that is, as a function of the applied electric field. Figure 6d shows the time trace of the thermally activated statistic switching of the conductance, while the applied voltage was slowly swept from 50 to 350 mV. Three enlarged views are shown at bias voltages around 180, 200 and 220 mV (Fig. 6a,d). Clearly, the population depends on the bias voltage. Averaging the discriminated signal over intervals of 7.5 s results in a smooth curve representing the voltage-dependent population of the ON state (*N*_ON_/(*N*_OFF_+*N*_ON_)), which is then translated into the energy difference between the ON and the OFF state according to equation (2), taking *T*=5.2 K from the thermometer reading. It can be seen that this energy difference scales linearly with the applied voltage as is expected for the energy of a dipole in an electric field. This clearly shows the role of the electric field and indicates that the transition is not caused by the tunnelling current. Furthermore, the slope of *a*=−26 meV V^−1^ allows to estimate the change in tilt of the dipole moment to ∼17°, when switching between the ON (*Φ*_ON_=48°) and OFF states (*Φ*_OFF_=65°; for details see Supplementary Note 12). The same experiment was performed on spiro B, where a change in tilt angle of *Φ*_OFF_−*Φ*_ON_=7° was found (Supplementary Fig. 18). Although the precise angle is expected to depend on the tip position, this difference can intuitively be explained by the shorter and therefore more rigid cantilever head group of this variant. In spite of the very low currents involved, we cannot exclude that the molecular temperature differs from the thermometer reading, which would influence the precise value of the tilt of the dipole.

Second, we extracted the energy difference between the open and closed molecular junction as a function of the distance between tip and sample that allows to deduce the involved force. Figure 6e shows the corresponding time trace of the current for a distance variation of 14 pm for spiro A at constant voltage. As this displacement is negligible in comparison to the overall tip–sample distance, the electric field can be assumed to be constant. Similar to the case of the voltage variation, we observe a continuous transition of the population from the ON state to the OFF state with increasing distance between tip and sample. The counter-intuitive increase in conductance while stretching the molecular junction might be explained by a release of mechanical stress and related changes in the orbital level alignment^41^,^42^,^43^. Strictly speaking, this experiment determines the energy difference between the ON and OFF configurations of the junction as a function of the *z* position of the tip, which corresponds, following classical mechanics, to the force that is needed to stretch the closed molecular junction. The slope of the fitted straight line is −0.9*k*_B_*T* pm^−1^. Assuming a temperature of *T*=5.2 K, this corresponds to −0.4 meV pm^−1^ which is, in other units, a force of 64 pN (42 pN for spiro B). In simulations of the stiffness of the molecule, we found values of the same order of magnitude (9.7 pN pm^−1^ for spiro A and 18.6 pN pm^−1^ for spiro B, see Supplementary Note 5 and Supplementary Fig. 9). This leads to a change of the potential energy by ±1.5 meV (±2.8 meV for spiro B) when compressing or stretching the molecular junction by 7 pm, in agreement with our experimental results. These stiffness values result in the experimentally observed forces when stretching the molecule by 6.6 pm in the case of spiro A (2.3 pm in the case of spiro B). However, the stiffness and the elastic deformation of tip, sample and in particular the bond between the molecule and the tip were not considered in these simulations. The nitrile–gold bond is expected to largely influence the stiffness of the overall molecular junction, as it is the predetermined breaking point. In addition, the bond between molecule and tip is weakened by the voltage applied in the experiments. As the thermally induced opening and closing of the molecular junction does not involve a change of the macroscopic tip–substrate distance, the force derived in this way does not contain related contributions from van der Waals forces that can amount up to 2 nN (refs 12, 14).

## Discussion

In contrast to typical AFM force measurements, only the energy variation related to the stretching of the molecular bridge is involved in our experiment. Thus, the method presented here allows to address exclusively the atomic forces involved in the junction formation provided that current-induced heating can be excluded. Given the low thermal energy of 0.4 meV at a temperature of 5.2 K, minute forces in the range of piconewtons can be extracted with high precision, but larger forces are out of reach due to too few thermally activated transitions.

Our results highlight the importance of atomic-scale control of the junction geometry to obtain reliable mechanical and electrical properties in future molecular devices. The very reliable control of the presented molecular toggle switch allowed us to demonstrate new experimental methods that reveal the subtle energy variations during contact formation on the nanoscale and that will be applicable to a wide range of single-molecule junctions.

## Methods

### Syntheses of the model compounds spiro A and spiro B

Our synthetic strategy used for the preparation of the 2,7,3′,6′-tetrasubstituted 9,9′-spirobifluorene tripodal derivatives **spiro A** and **spiro B** is outlined in Supplementary Fig. 1. The first modular platform based on a rigid 9,9′-spirobifluorene with three acetyl protected thiol groups in the positions 2, 3′ and 6′ exposing a *para*-cyanophenylethynyl rod in the position 7 (**spiro A**) was reported recently^27^. Our synthetic approach is based on a metal-halogen exchange reaction of the 2-iodobiphenyl derivative and its subsequent reaction with 2,7-disubstituted fluoren-9-one to afford the carbinol. Further electrophilic cyclization and separation of regioisomers provided the corresponding 2,7,3′,6′-tetrasubstituted 9,9′-spirobifluorene **1** as the key intermediate. This previously prepared spirobifluorene derivative **1** (ref. 27) was also employed for a subsequent synthesis of a shorter derivative spiro B. To introduce the third alkylsulfanyl group, spirobifluorene derivative **1** was treated with 2-(trimethylsilyl)ethanthiol in the presence of Pd_2_(dba)_3_, Xantphos and Hünig's base to provide the desired tripodal spirobifluorene **2** in 75% yield (Supplementary Note 1). Subsequent cyanation of bromo derivative **2** afforded nitrile derivative **3** in 79% yield (Supplementary Note 2). Final transprotection of the thiol was successfully performed using AgBF_4_ and acetyl chloride in dichloromethane to obtain the desired tripodal thioacetate spiro B in 95% yield (Supplementary Note 3). The presence of terminal thioacetate groups allows the target spirobifluorene molecules **spiro A** and **spiro B** to bind to gold surfaces. The acetyl works as a labile thiol-protecting group, and can mildly and efficiently be cleaved by thermal annealing upon deposition of molecules on a gold surface.

All starting materials and reagents were obtained from commercial suppliers and used without further purification. Thin-layer chromatography was performed on Silica gel 60 F_254_ plates, spots were detected by fluorescence quenching under ultraviolet light at 254 nm and/or staining with appropriate solutions (anisaldehyde, phosphomolybdic acid, KMnO_4_). Column chromatography was performed on Silica gel 60 (0.040–0.063 mm). All experimental manipulations with anhydrous solvents were carried out in flame-dried glassware under inert atmosphere of argon. Degassed solvents were obtained by three cycles of the freeze–pump–thaw. Dioxane was dried and distilled from sodium/benzophenone under argon atmosphere. Dichlormethane was dried and distilled from CaH_2_ under argon atmosphere. *S,S*′*,S*″-[7-(4-Cyanophenylethynyl)-9,9′-spirobifluorene-2,3′,6′-triyl] tris(thioacetate) (**spiro A**)^27^ and 2-bromo-7-iodo-3′,6′-bis[2-(trimethylsilyl)ethylsulfanyl]-9,9′-spirobifluorene **(1)** were prepared according to the published procedure^27^.

All nuclear magnetic resonance (NMR) spectra were recorded at 25 °C in CDCl_3_. ^1^H NMR (500.16 MHz) spectra were referenced to the solvent residual proton signal (CDCl_3_, *δ*_H_=7.24 p.p.m.). ^13^C NMR (125.78 MHz) spectra with total decoupling of protons were referenced to the solvent (CDCl_3_, *δ*_C_=77.23 p.p.m.). For correct assignment of both ^1^H and ^13^C NMR spectra, the ^1^H–^1^H correlation spectroscopy (COSY), ^13^C DEPT-135, heteronuclear single quantum coherence (HSQC) and heteronuclear multiple bond correlation (HMBC) experiments were performed. Electron ionization mass spectra (EI MS) were recorded with a gas chromatography mass spectrometry (GC/MS) instrument (samples were dissolved in diethyl ether, chloroform or introduced directly using direct injection probes (DIP) and direct exposure probes (DEP)), and *m/z* values are given along with their relative intensities (%) at an ionizing voltage of 70 eV. Infrared spectra were measured in KBr pellets. Analytical samples were dried at 40–100 °C under reduced pressure (10^−2^ mbar). Elemental analyses were obtained using an elemental analyser. NMR spectra of the compounds **2**, **3** and **spiro B** are displayed in Supplementary Figs 2–7.

### Electronic structure calculations

Atomistic simulations are performed to support and help interpret the experimental observations. Electronic structure calculations and related geometry optimizations are carried out by applying the quantum chemistry software package TURBOMOLE 6.6 (ref. 44). All the calculations were performed within DFT using the exchange-correlation functional perdew burke-ernzerhof (PBE)^45^,^46^,^47^,^48^ and the def-SV(P) basis set^49^,^50^,^51^, which is of split-valence quality with polarization functions on all non-hydrogen atoms.

To analyse the electron transport through the spiro molecules on an Au(111) surface (Fig. 3), we followed the procedure described in refs 52, 53. The molecular geometry was first optimized on the surface, keeping all of the gold atoms fixed. Subsequently, the STM tip was connected to the head of the molecule. The Au pyramid, chosen as the STM tip, and the surface represent semi-infinite electrodes. They are oriented along the (111) direction, which coincides with the transport direction or *z* axis.

The theoretical investigation of the quantum transport properties of complex molecules remains challenging because of the large number of atoms involved and the infinite, non-periodic geometry of the system. DFT is one of the few *ab initio* electronic structure methods that can handle the hybrid metal–molecule–metal contacts. On the other hand, due to self-interaction errors in the standard exchange-correlation functional and missing image charge effects, DFT-based methods have difficulties to accurately describe the energy gap and level alignment of molecules at surfaces. This can be improved by adding a self-energy correction, resulting in the DFT+Σ method^33^,^34^,^35^. The main difference in the electronic structure between DFT and DFT+Σ for a given contact geometry is typically a pronounced increase of the gap between the highest occupied molecular orbital and lowest unoccupied molecular orbital by several eVs that arise from a (almost symmetric) decrease of the occupied energy levels and increase of the unoccupied energy levels with respect to the Fermi energy *E*_F_ of the metal electrodes (see Fig. 3 and its discussion). Details regarding our DFT+Σ implementation can be found in ref. 35, and for more information on our particular DFT+Σ calculations, see Supplementary Note 7 and Supplementary Fig. 11.

Using the electronic structure of DFT and DFT+Σ as input, the transmission *τ*(*E*) as a function of energy is calculated using the Landauer-Büttiker formalism expressed in terms of nonequilibrium Green's functions^32^. In the Landauer-Büttiker approach, the stationary current is given as


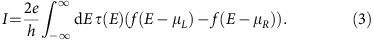


For the linear conductance one obtains


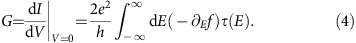


### STM experiments

STM measurements were carried out in a home-built STM in UHV at a temperature of 5.2 K. The STM tip was prepared by chemical etching of a tungsten wire and by repeated dipping into the gold surface. The Au(111) single-crystal surface was cleaned by several cycles of Ar^+^ sputtering and annealing to 700 K. Spirobifluorene molecules were sprayed onto the surface through a pulse valve at a pressure of ∼1 mbar. After the deposition, the sample was transferred into the UHV chamber, annealed at 410 K for 1 h and subsequently transferred to the STM. The voltage is applied to the sample.

### Data availability

The data that support the findings of this study are available from the authors on reasonable request, see author contributions for specific data sets.

## Additional information

**How to cite this article:** Gerhard, L. *et al*. An electrically actuated molecular toggle switch. *Nat. Commun.* **8,** 14672 doi: 10.1038/ncomms14672 (2017).

**Publisher's note**: Springer Nature remains neutral with regard to jurisdictional claims in published maps and institutional affiliations.

## Supplementary Material

Supplementary Information Supplementary Figures, Supplementary Notes and Supplementary References

## Figures and Tables

**Table 1:** Conductance of the molecular junction.

	a	b	c	d	e	f
*G*_DFT_	1.6 × 10^−3^	2.1 × 10^−3^	5.1 × 10^−5^	3.9 × 10^−5^	5.2 × 10^−3^	4.9 × 10^−3^
*G*_DFT+Σ_	8.0 × 10^−4^	5.0 × 10^−4^	2.1 × 10^−5^	1.4 × 10^−5^	2.5 × 10^−3^	2.8 × 10^−3^
Experiment	5 × 10^−6^				4 × 10^−4^	

Computed conductance of the configurations shown in Fig. 3 using DFT and DFT+Σ methods in comparison with experimental values. Conductances are given in units of *G*_0_=2*e*^2^/*h*.

**Table 2:** Calculated dipole moments.

**Benzonitrile**	**Spiro A**	**Spiro A-rotated ring**	**Spiro B**
4.33 D	6.45 D	5.77 D	5.06 D
