# Supplementary material for: An electrically actuated molecular toggle switch
Source: Nat Commun. 2017 Mar 9;8:14672. doi: 10.1038/ncomms14672 (PMC5347093; doi:10.1038/ncomms14672)
Supplement: Supplementary Information — Supplementary Figures, Supplementary Notes and Supplementary References [file ncomms14672-s1.pdf]

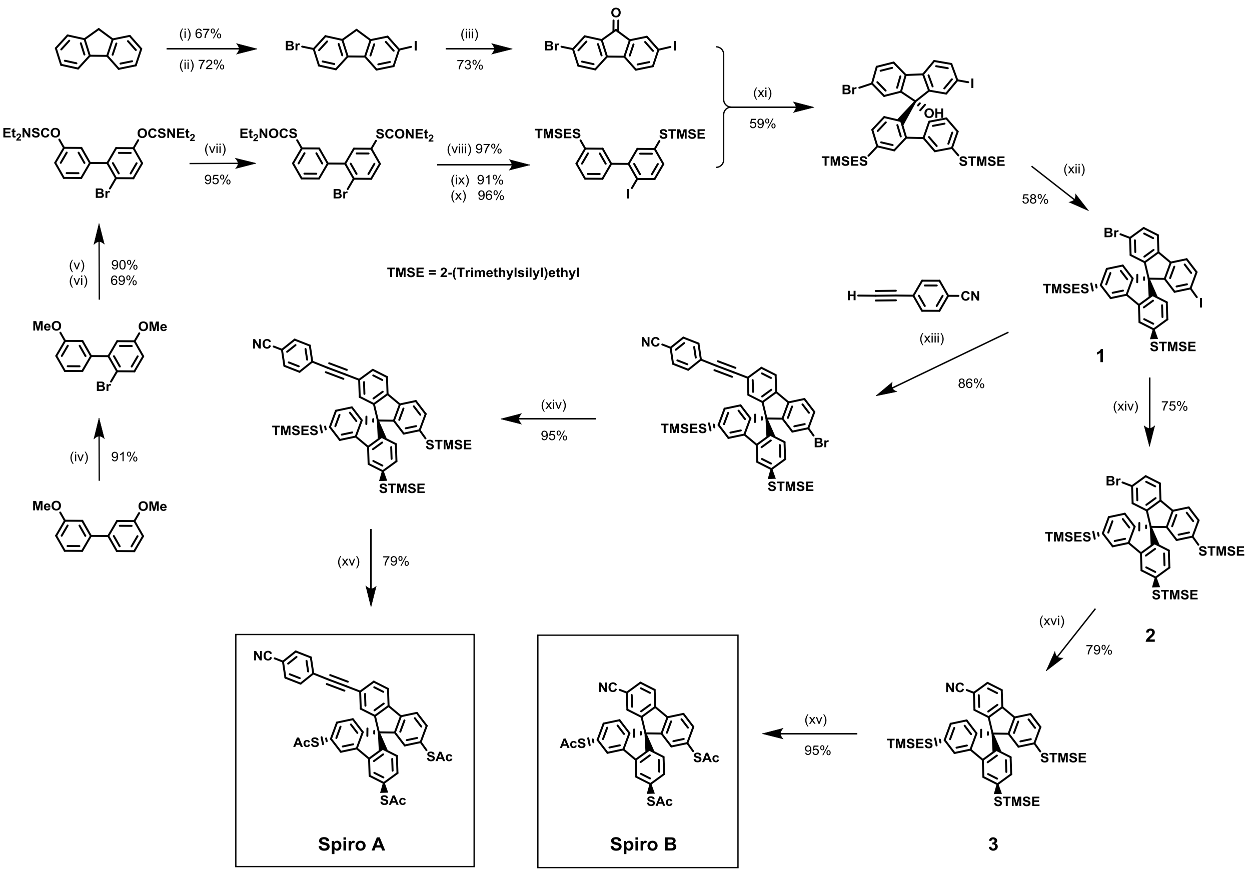

Supplementary Figure 1. **Syntheses of spirobifluorene derivatives spiro A and spiro B.** Reagents and conditions: (i) NBS, propylene carbonate; (ii) I_2, KIO_3, H^+; (iii), CrO_3, AcOH, Ac_2O; (iv) NBS, CH_3CN; (v) BBr_3, CH_2Cl_2; (vi) NaH, Et_2NCSCl, DMF, Δ; (vii) Ph_2O, Δ; (viii) a) KOH, MeOH, b) H^+; (ix) vinyltrimethylsilane, AIBN, Δ; (x) a) *n*-BuLi, THF, b) I_2, THF; (xi) *iso*-PrMgCl·LiCl, THF, -40 °C; (xii) AcOH, HCl; (xiii) $PdCl_2(PPh_3)_2$, CuI, Et_3N; (xiv) $TMS(CH_2)_2SH$, $Pd_2(dba)_3$, XPhos, *iso*-Pr_2EtNH, dioxane, Δ; (xv) $AgBF_4$, AcCl, CH_2Cl_2; (xvi) $Zn(CN)_2$, CuCN, DMF, Δ.

Supplementary Note 1. 2-BROMO-3',6',7-TRIS[2-(TRIMETHYLSILYL)ETHYLSULFANYL]-9,9'-SPIROBIFLUORENE (2)

A dry 15 ml pressure tube was charged with regioisomerically pure spirobifluorene **1** [1] (300 mg, 382 μmol), Xantphos (12 mg, 21 μmol), $Pd_2(dba)_3$ (10 mg, 10 μmol), and dry dioxane (7 ml). The tube was evacuated under vacuum and refilled with argon three times. Then *N,N*-diisopropylethylamine (149 mg, 196 μl, 1.15 mmol), and 2-(trimethylsilyl)ethanethiol

(56 mg, 67 μl, 420 μmol) were added under argon and the tube was quickly capped. The reaction mixture was heated at 80 °C for 15 h. The completion of the reaction was checked by TLC (hexane:EtOAc = 40:1). After cooling, the reaction mixture was diluted with diethyl ether (20 ml), filtered through a pad of silica gel (20 g, diethyl ether), and the filtrate was concentrated in vacuo. The crude product was purified by column chromatography on silica gel (300 g, hexane:EtOAc = 40:1) to provide the title compound **2** (226 mg) as a colorless oil in 75% yield (R_f= 0.29, hexane:EtOAc = 40:1). ^{1}H NMR (500 MHz, $CDCl_3$) δ ppm: -0.11 (s, 9H, $CH_3^{(\to 7)}$), 0.06 (s, 18H, $CH_3^{(\to 3')}$, $CH_3^{(\to 6')}$), 0.75 (m, 2H, $CH_2Si^{(\to 7)}$), 0.99 (m, 4H, C $H_2Si^{(\to 3')}$, $CH_2Si^{(\to 6')}$), 2.77 (m, 2H, $SCH_2{}^{(\to 7)}$), 3.02 (m, 4H, $SCH_2{}^{(\to 3')}$, $SCH_2{}^{(\to 6')}$), 6.59 (d, J = 1 Hz, 1H, C^8H), 6.61 (d, J = 8 Hz, 2H, $C^{1'}$H, $C^{8'}$H), 6.81 (d, J = 1 Hz, 1H, C^1H), 7.05 (dd, J = 8 Hz, J = 1 Hz, 2H, $C^{2'}$H, $C^{7'}$H), 7.26 (dd, J = 8 Hz, J = 1 Hz, 1H, C^6H), 7.44 (dd, J = 8 Hz, J = 1 Hz, 1H, C^3H), 7.60 (d, J = 8 Hz, 1H, C^4H), 7.67 (d, J = 8 Hz, 1H, C^5H), 7.71 (d, J = 1 Hz, 2H, $C^{4'}$ H, $C^{5'}$H). ^{13}C NMR (125.8 MHz, $CDCl_3$) δ ppm: -1.7 ($CH_3^{(\to 7)}$), -1.5 ($CH_3^{(\to 3')}$, $CH_3^{(\to 6')}$), 17.0 ($CH_2Si^{(\to 7)}$), 17.2 ($CH_2Si^{(\to 3')}$, $CH_2Si^{(\to 6')}$), 29.5 ($SCH_2{}^{(\to 7)}$), 30.0 ($SCH_2{}^{(\to 3')}$, $SCH_2{}^{(\to 6')}$), 65.2 (C^9), 120.5 ($C^{4'}$H, $C^{5'}$H), 120.6 (C^5H), 121.3 (C^4H), 121.4 (C^2), 124.3 (C^8H), 124.6 ($C^{1'}$H, $C^{8'}$H), 127.5 (C^1H), 128.7 (C^6H), 128.9 ($C^{2'}$H, $C^{7'}$H), 131.3 (C^3H), 137.6 (C^7), 137.8 ($C^{3'}$, $C^{6'}$), 138.5 (C^{12}), 140.5 (C^{11}), 142.1 ($C^{11'}$, $C^{12'}$), 145.8 ($C^{10'}$, $C^{13'}$), 149.1 (C^{13}), 150.5 (C^{10}). IR (KBr) ν cm^{-1} 2951 (m) and 2918 (m, $\nu_{as}(CH_2, CH_3)$), 2851 (w, $\nu_s(CH_2, CH_3)$), 1598 (w) and 1561 (w, ν(CC)), 1452 (m), 1415 (m), 1248 (s, $\delta_s(CH_3)$, TMS), 1162 (m), 1125 (w), 1073 (w), 1009 (w), 958 (w), 858 (vs) and 839 (vs, $\delta_{as}(CH_3)$, TMS), 752 (m), 693 (m, $v_{as}(SiC_3)$, TMS), 637 (w). EI MS m/z (%) 792 (2), 790 (2, M^+), 708 (2), 706 (2), 101 (3), 86 (60), 84 (100), 73 (21), 51 (24), 49 (75). Anal. calcd. for $C_{40}H_{51}BrS_3Si_3$ (792.19): C, 60.65; H, 6.49. Found: C, 60.98; H, 6.65.

Supplementary Note 2. 3',6',7-TRIS[2-(TRIMETHYLSILYL)ETHYLSULFANYL]-9,9'-SPIROBIFLUORENE-2-CARBONITRILE (3)

A dry 25 ml round bottom flask was charged with 2-bromospirobifluorene **2** (0.20 g, 0.25 mmol), zinc cyanide (29 mg, 0.25 mmol), copper(I) cyanide (20 mg, 0.22 mmol), and anhydrous DMF (15 ml) under argon. The suspension was purged with argon for 15 min before $Pd(PPh_3)_4$ (29 mg, 0.025 mmol) was added. The flask was then sealed and stirred

at 150 °C for 20 h. The completion of the reaction was checked by TLC (hexane:EtOAc = 20:1). After cooling to room temperature, the resulting solution was quenched with Na_2 CO_3 (10%, 60 ml) and ethyl acetate (100 ml) was added. The aqueous layer was washed with ethyl acetate (2 × 60 ml). The combined organic layer was washed with brine (100 ml), dried over $MgSO_4$ and filtered. All volatiles were removed under reduced pressure and a light yellow residue was purified by column chromatography on silica gel (300 g) in the mixture of hexane/EtOAc (40:1) to get 146 mg of **3** as a colorless oil in 79% yield (R_f= 0.24, hexane:EtOAc = 15:1). ^{1}H NMR (500 MHz, $CDCl_3$) δ ppm: -0.10 (s, 9H, $CH_3^{(\rightarrow 7)}$), 0.07 (s, 18H, $CH_3^{(\rightarrow 3')}$, $CH_3^{(\rightarrow 6')}$), 0.76 (m, 2H, $CH_2Si^{(\rightarrow 7)}$), 0.99 (m, 4H, $CH_2Si^{(\rightarrow 3')}$, $CH_2Si^{(\rightarrow 6')}$), 2.80 (m, 2H, $SCH_2^{(\rightarrow 7)}$), 3.03 (m, 4H, $SCH_2^{(\rightarrow 3')}$, $SCH_2^{(\rightarrow 6')}$), 6.59 (d, J = 8 Hz, 2H, $C^{1'}$H, $C^{8'}$H), 6.61 (d, J = 1 Hz, 1H, C^8H), 6.96 (d, J = 1 Hz, 1H, C^1H), 7.06 (dd, J = 8 Hz, J = 1 Hz, 2H, $C^{2'}$H, $C^{7'}$H), 7.29 (dd, J = 8.5 Hz, J = 1.5 Hz, 1H, C^3H), 7.61 (dd, J = 8 Hz, J = 1 Hz, 1H, C^6H), 7.73 (d, J = 1.5 Hz, 2H, $C^{4'}$H, $C^{5'}$H), 7.75 (d, J = 8 Hz, 1H, C^4H), 7.81 (d, J = 8 Hz, 1H, C^5H). ^{13}C NMR (125.8 MHz, $CDCl_3$) δ ppm: -1.7 ($CH_3^{(\rightarrow 7)}$), -1.5 ($CH_3^{(\rightarrow 3')}$, $CH_3^{(\rightarrow 6')}$), 16.8 ($CH_2Si^{(\rightarrow 7)}$), 17.1 ($CH_2Si^{(\rightarrow 3')}$, $CH_2Si^{(\rightarrow 6')}$), 28.9 ($SCH_2^{(\rightarrow 7)}$), 29.9 ($SCH_2^{(\rightarrow 3')}$, $SCH_2^{(\rightarrow 6')}$), 65.1 (C^9), 110.7 (C^2), 119.2 (CN), 120.45 (C^4H), 120.53 ($C^{4'}$H, $C^{5'}$H), 121.5 (C^5H), 123.5 (C^8H), 124.4 ($C^{1'}$H, $C^{8'}$H), 127.9 (C^1H), 128.2 (C^6H), 128.8 ($C^{2'}$H, $C^{7'}$H), 132.4 (C^3H), 137.2 (C^{12}), 138.2 ($C^{3'}$, $C^{6'}$), 139.9 (C^7), 142.1 ($C^{11'}$, $C^{12'}$), 144.8 ($C^{10'}$, $C^{13'}$), 145.9 (C^{11}), 149.3 (C^{10}), 150.0 (C^{13}). IR (KBr) ν cm^{-1} 3054 (w, ν(=CH)), 2950 (s) and 2918 (m, ν_{as}(CH_2,CH_3)), 2895 (m, ν_s(CH_2,CH_3)), 2223 (m, ν(C≡N)), 2212 (w, ν(C≡C)), 1599 (m) and 1564 (w, ν(CC)), 1460 (m), 1414 (m), 1248 (s, δ_s(CH_3), TMS), 1164 (m), 1125 (vw), 1104 (vw), 1074 (w), 1009 (m), 960 (vw), 857 (vs) and 839 (vs, δ_{as}(CH_3), TMS), 752 (m), 694 (m, v_{as}(SiC_3), TMS), 639 (m), 529 (w). EI MS *m/z* (%) 737 (3, M^+), 709 (3), 681 (3), 653 (3), 86 (60), 84 (91), 73 (32), 49 (100). Anal. calcd. for $C_{41}H_{51}NS_3Si_3$ (738.30): C, 66.70; H, 6.93; N, 1.90. Found: C, 66.95; H, 6.81; N, 1.87.

Supplementary Note 3. ***S,S',S"*-(2-CYANO-9,9'-SPIROBIFLUORENE-3',6',7-TRIYL) TRIS(THIOACETATE) (SPIRO B)**

In a 30 ml Schlenk flask, spirobifluorene **3** (100 mg, 135 μmol) was dissolved in the mixture of dry dichloromethane (10 ml) and acetyl chloride (1 ml), and put under argon. The solution was cooled to 0 °C and then treated with $AgBF_4$ (160 mg, 0.82 mmol). A

violet suspension was stirred at 0 °C for 8 h. The completion of the reaction was checked by TLC (hexane:EtOAc = 3:1). The reaction mixture was diluted with dichloromethane (50 ml) at 0 °C then slowly quenched with water (10 ml), and the white precipitate was filtered through a pad of Celite (CH_2Cl_2). The filtrate was extracted with water (30 ml), brine (30 ml), and dried with magnesium sulfate. The solution was passed through a pad of silica gel (10 g, CH_2Cl_2), and the filtrate was concentrated in vacuo. The crude product was purified by column chromatography on silica gel (200 g, hexane:EtOAc = 4:1) to provide the target thioacetate **spiro B** (72 mg) as a white foamy solid in 95% yield (R_f= 0.22, hexane:EtOAc = 4:1). ^{1}H NMR (500 MHz, $CDCl_3$) δ ppm: 2.32 (s, 3H, $CH_3^{(\to 7)}$), 2.45 (s, 6H, $CH_3^{(\to 3')}$, $CH_3^{(\to 6')}$), 6.73 (d, J = 8 Hz, 2H, $C^{1'}$H, $C^{8'}$H), 6.83 (d, J = 1 Hz, 1H, C^8H), 7.01 (d, J = 1 Hz, 1H, C^1H), 7.18 (dd, J = 8 Hz, J = 1.5 Hz, 2H, $C^{2'}$H, $C^{7'}$H), 7.50 (dd, J = 8 Hz, J = 1.5 Hz, 1H, C^6H), 7.67 (dd, J = 8 Hz, J = 1 Hz, 1H, C^3H), 7.88 (d, J = 1 Hz, 2H, $C^{4'}$H, $C^{5'}$H), 7.90 (d, J = 8 Hz, 1H, C^4H), 7.91 (d, J = 8 Hz, 1H, C^5H). ^{13}C NMR (125.8 MHz, $CDCl_3$) δ ppm: 30.4 ($CH_3^{(\to 7)}$), 30.5 ($CH_3^{(\to 3')}$, $CH_3^{(\to 6')}$), 65.5 (C^9), 112.0 (C^2), 118.8 (CN), 121.3 (C^4H), 121.9 (C^5H), 124.9 ($C^{1'}$H, $C^{8'}$H), 126.9 ($C^{4'}$H, $C^{5'}$H), 128.2 (C^1H), 128.9 ($C^{3'}$, $C^{6'}$), 130.0 (C^7), 130.3 (C^8H), 132.8 (C^3H), 134.6 ($C^{2'}$H, $C^{7'}$H), 135.3 (C^6H), 141.0 (C^{12}), 142.1 ($C^{11'}$, $C^{12'}$), 145.3 (C^{11}), 147.9 ($C^{10'}$, $C^{13'}$), 149.02 (C^{10}), 149.04 (C^{13}), 193.4 ($CO^{(\to 7)}$), 193.8 ($CO^{(\to 3')}$, $CO^{(\to 6')}$). IR (KBr) ν cm^{-1} 2925 (s, $\nu_{as}(CH_3)$), 2859 (m, $\nu_s(CH_3)$), 2225 (m, ν(C≡N)), 1707 (vs, ν(C=O)), 1608 (m, ν(CC)), 1494 (s), 1455 (s), 1405 (m), 1385 (s), 1352 (m), 1262 (w), 1119 (vs), 1050 (w), 949 (m), 876 (m), 824 (s), 687 (w), 640 (m), 615 (s), 549 (w), 417 (w). EI MS m/z (%) 563 (1, M^+), 521 (2), 479 (2), 437 (3), 404 (2), 370 (1), 86 (100), 51 (90), 49 (85), 47 (44). Anal. calcd. for $C_{32}H_{21}NO_3S_3$ (563.70): C, 68.18; H, 3.76; N, 2.48. Found: C, 68.37; H, 3.68; N, 2.53.

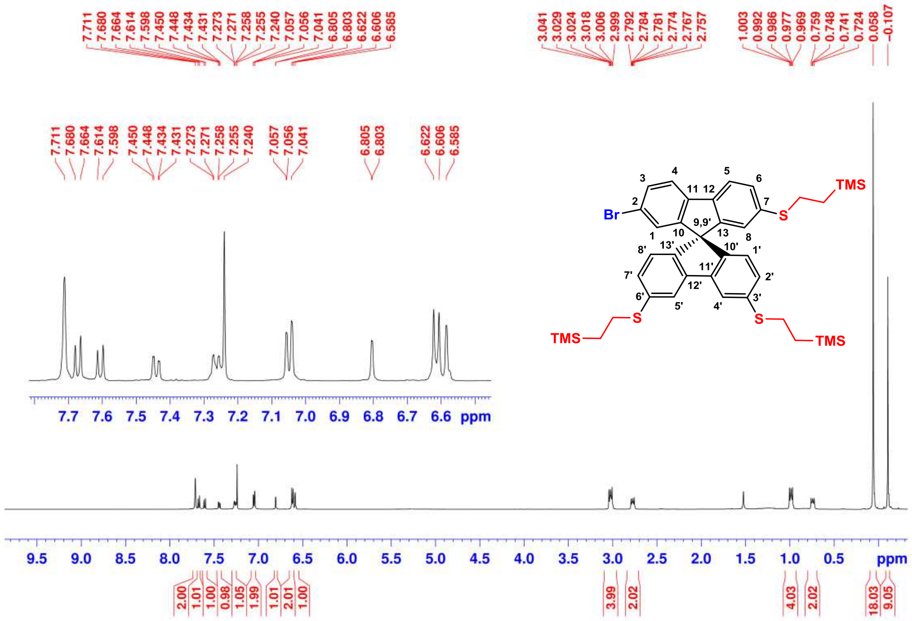

Supplementary Figure 2. ^{1}H NMR spectrum (500 MHz, $CDCl_3$) of **2**.

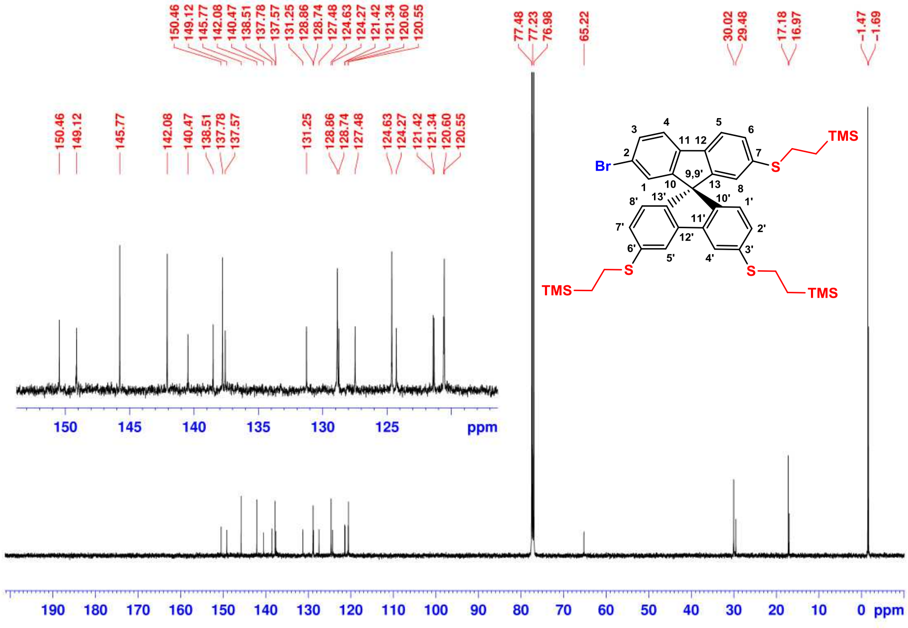

Supplementary Figure 3. ^{13}C NMR spectrum (125.8 MHz, $CDCl_3$) of **2**.

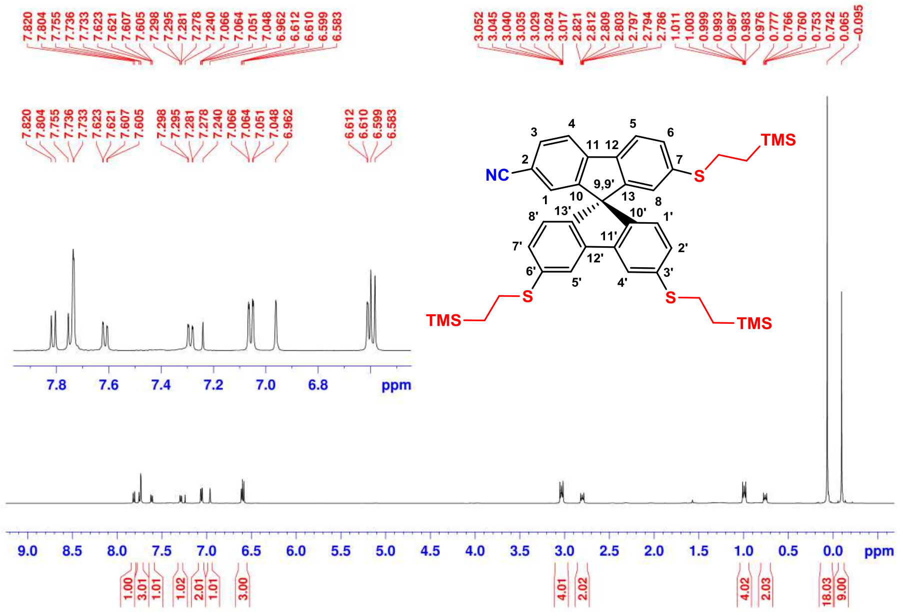

Supplementary Figure 4. ^{1}H NMR spectrum (500 MHz, $CDCl_3$) of **3**.

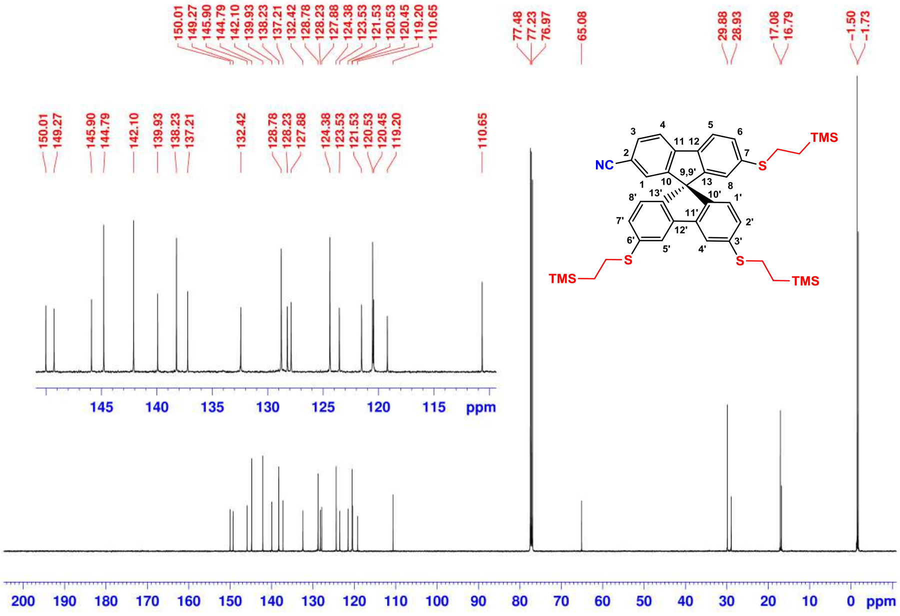

Supplementary Figure 5. ^{13}C NMR spectrum (125.8 MHz, $CDCl_3$) of **3**.

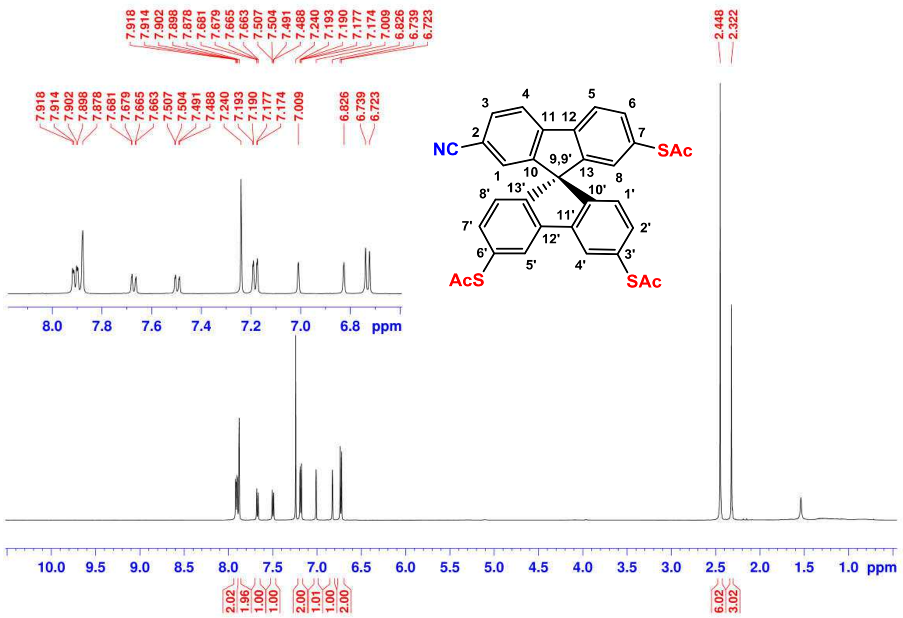

Supplementary Figure 6. ^{1}H NMR spectrum (500 MHz, $CDCl_3$) of **spiro B**.

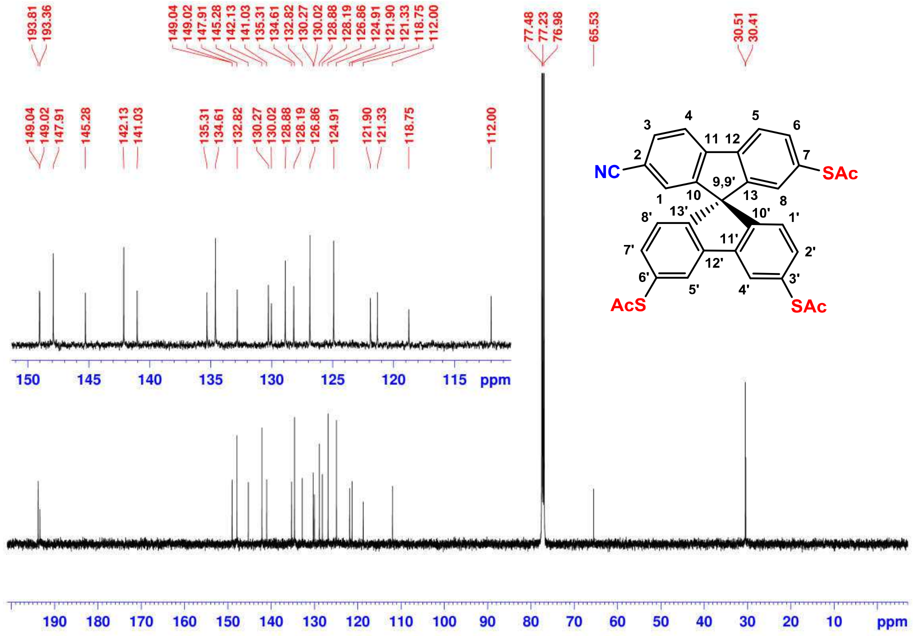

Supplementary Figure 7. ^{13}C NMR spectrum (125.8 MHz, $CDCl_3$) of **spiro B**.

Supplementary Note 4. GAS-PHASE RELAXATION OF THE TRIPODAL MOLECULES

The optimized geometries of the spiro A and spiro B molecules were determined in the gas-phase. These structures are shown in Supplementary Fig. 8 together with an additional variant of spiro A with a rotated phenyl ring at the head of the molecule (Supplementary Fig. 8b). Rotating the head phenyl group by 90° leads to an energy increase of around 0.2 eV, as compared to the ground-state configuration in Supplementary Fig. 8a.

In order to compare to the angles determined in the ON and OFF states, discussed in the main text, we studied the angles between the surface normal of the plane spanned by the three sulfur atoms and the N-S axis, Φ_1, or the C-N axis, Φ_2, as indicated in Supplementary Fig. 8b. The corresponding values are listed in Supplementary Table 1. We henceforth assume that the surface normal coincides with the z-direction and that the plane of the three sulfur atoms at the legs is the x-y-plane, where the y-axis is oriented along the molecular backbone. X-ray measurements of a crystal of the molecular precursor of spiro A and B showed an angle of $\Phi_{\mathrm{ON}} = 52.8°$ [1].

Angle	Spiro A	Spiro A - rotated ring	Spiro B
Φ_1 (deg)	46.1	46.1	43.4
Φ_2 (deg)	52.1	52.1	52.4
Φ_{dip} (deg)	56.2	57.5	54.4

Supplementary Table 1. Angles Φ_1 and Φ_2, as extracted from Supplementary Fig. 8, and the angle of the dipole moment, all with regard to the normal (z-direction) of the plane spanned by the three sulfur atoms (x-y-plane).

Supplementary Note 5. SIMULATED FORCE CONSTANTS

The elastic deformation of a molecular junction is described to lowest order by Hooke's law, which states that the small displacements of the atoms from the equilibrium positions are proportional to the applied force. We simulated the compression or stretching of the molecular junction by slight changes (in steps of 0.02 Å) of the position of the nitrogen atom in the z-direction. In the procedure, the sulfur atoms and the nitrogen atom were kept

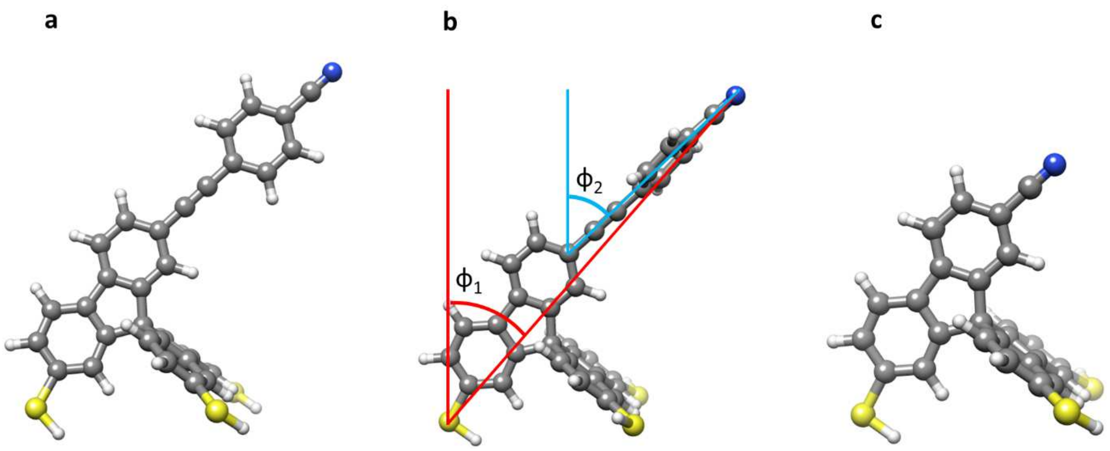

Supplementary Figure 8. **Gas-phase relaxation of the tripodal molecules.** Optimized geometries in the gas phase for **a**, spiro A, **b**, spiro A with the phenyl ring at its head rotated by 90°, referred to as spiro A - rotated ring, and **c**, spiro B. The definition of the angles Φ_1 and Φ_2 is shown in panel **b**.

fixed, while the rest of the molecule was relaxed, as shown schematically in Supplementary Fig. 9a. The total energy versus the vertical displacement of the nitrogen atom is plotted in Supplementary Fig. 9b,c for spiro A and B, and the force constants are extracted by fitting a parabola to the energy-displacement curve. With this approach, we find a vertical molecular stiffness of k = 9.7 pN pm^{-1} for spiro A, while spiro B is stiffer with k = 18.6 pN pm^{-1}, as expected due to the shorter lever.

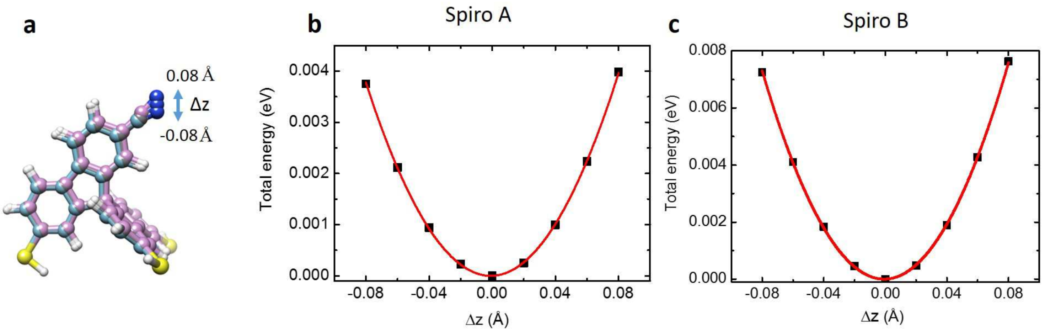

Supplementary Figure 9. **Calculation of the molecular stiffness.** **a**, Sketch of the procedure used for calculating the molecular stiffness, exemplified here for spiro B. The total energy versus vertical displacement of the N atom and the resulting stiffness of **b**, spiro A, and **c**, spiro B as extracted from a fit with a parabola.

Supplementary Note 6. SCANNING SIMULATIONS FOR BOND ENERGETICS

The z_{OFF}-z_{ON}-hysteresis map of spiro B is asymmetric with respect to a displacement of the tip along the molecular backbone, as shown in Supplementary Fig. 15. To inspect this, we calculated total energies for many positions of the STM gold tip in the y-z-plane, i.e., when it is placed above the backbone and in front of the molecular head as well as for different vertical distances from the molecule. In the simulations, we use a gold tip of 20 atoms and keep its two outermost gold layers fixed, defining the position of the tip. The system, consisting of tip and molecule, was subsequently relaxed by keeping, in addition to the outermost two Au layers, also the sulfur atoms fixed to mimic an infinitely stiff surface. The total energy of the system was calculated after relaxation of the remaining free atoms of the tip and molecule. This procedure is justified by the fact that we observe essentially no hysteresis in the case that the three S atoms are fixed (see also Fig. 2c), and the energy is hence unique. Starting from a position close to the smallest total energy, the two outermost gold layers were moved in steps of 0.1 Å horizontally either to the left (-y) or right (+y) or vertically down (-z) or up (+z). The total movement of the tip is 3 Å along the y-axis and 2 Å along the z-axis (see Supplementary Fig. 10).

In order to examine the effect of tip shape, we considered two configurations (see Supplementary Fig. 10), where the tip was rotated by 180° around the z-axis. With the tip positioned in front of the nitrile or cyano group, in one case either the edge of the pyramid-shaped tip or the lateral face are oriented towards the nitrogen atom. In agreement with the experiment, we observe that the total energy maps reflect the asymmetry with respect to a displacement of the tip along the molecular backbone (Supplementary Fig. 10a,b). The highest binding energies between tip and molecule occur in front of the nitrogen atom. The tip orientation does not affect this behavior. To quantify the binding, also the bond length between the Au tip atom and the nitrogen can be used. It shows a behavior similar to those of the total energy for both configurations (Supplementary Fig. 10c,d).

Supplementary Note 7. DETAILS OF DFT+Σ CALCULATIONS

In this section, we present further details of our DFT+Σ calculations. We use them in order to better describe the quasiparticle energies and the level alignment in our metal-

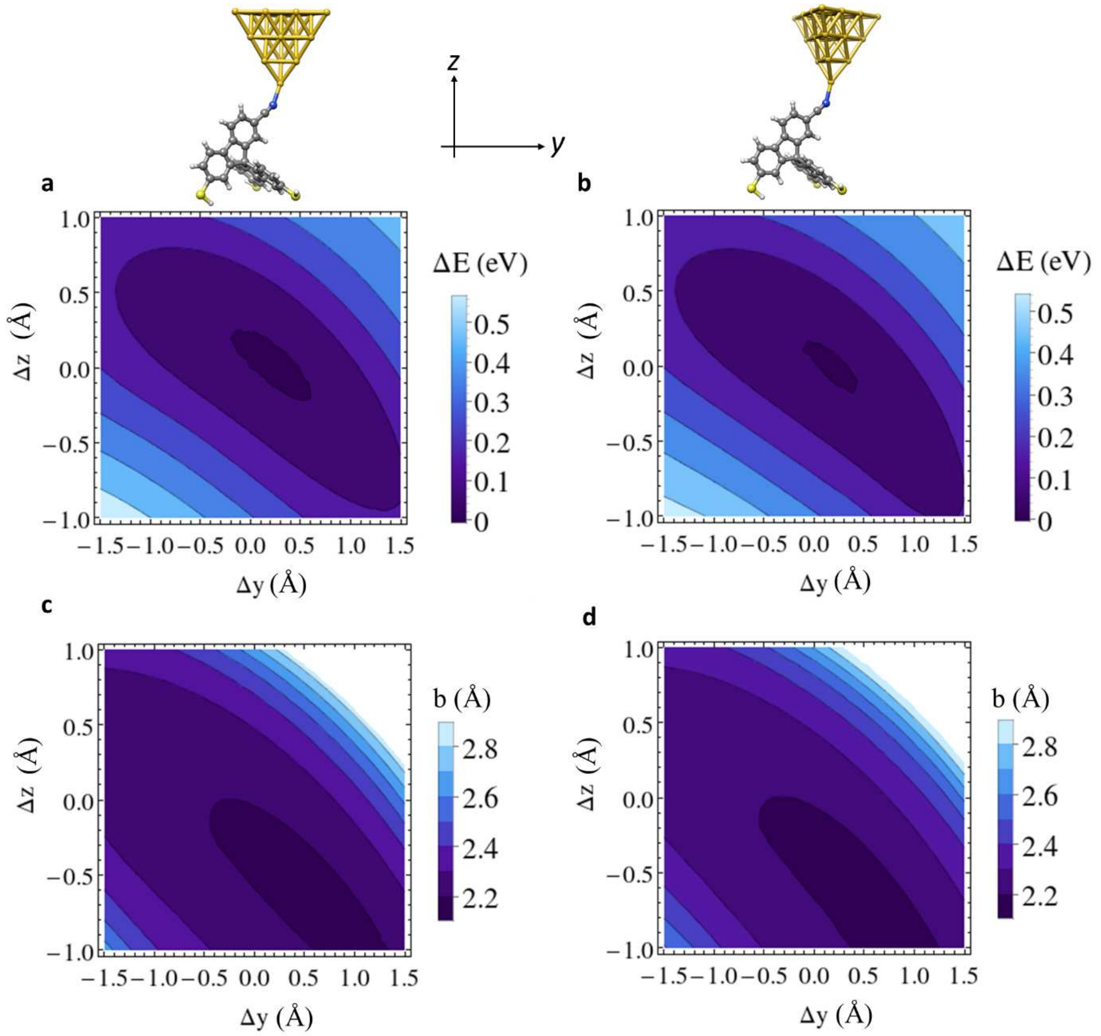

Supplementary Figure 10. **Simulation of the molecule-tip binding for spiro B, exploring different tip positions. a,b**, Maps of the total energy. **c,d**, Maps of the bond length.

molecule-metal junctions, improving at the same time the predicted charge transport properties.

It is known that DFT with semilocal exchange-correlation functionals, as used here, tends to underestimate HOMO-LUMO gaps of molecules and does not capture nonlocal surface polarization effects. The latter arise, when a molecule is brought in contact with a metal, where image-charge interactions shift the energy of the occupied states up and the virtual (unoccupied) ones down. The DFT+Σ approach aims at correcting the DFT-based energy levels of the molecule, and has been reported to lead to a better agreement between experimental and theoretical conductance values [2–4].

In the established approach, the screening of the metallic electrodes is described classically as the interaction of charge densities on the molecule, which we model through point-like Mulliken charges, with two perfectly conducting semiinfinite surfaces. Following Ref. [4], we use the charge distribution of the HOMO to calculate the image-charge correction, $\Delta_{\mathrm{occ}} = -\Delta_{\mathrm{HOMO}}$, for all the occupied states, while the LUMO charge distribution is used to determine the correction $\Delta_{\mathrm{virt}} = \Delta_{\mathrm{LUMO}}$ for the virtual or unoccupied states. The position of the image plane is chosen to be 1.47 Å outside of the first unrelaxed gold layer of the left and right electrode. This value corresponds to those for a perfectly flat surface and constitutes an approximation especially at the tip side, since we neglect the screening effects due to the apex atoms. Finally, the energy shift applied to all the occupied states is given by $\Sigma_{\mathrm{occ}} = -\mathrm{IP} - \epsilon_{\mathrm{H}} + \Delta_{\mathrm{occ}}$ and the corresponding one for the unoccupied states by $\Sigma_{\mathrm{virt}} = -\mathrm{EA} - \epsilon_{\mathrm{L}} + \Delta_{\mathrm{virt}}$. Here, ϵ_{H} and ϵ_{L} are the Kohn-Sham HOMO and LUMO energies of the molecule, and IP and EA are the ionization potentials and electron affinities, respectively, as obtained from ΔSCF total energy calculations of uncharged and singly charged molecules [4].

Complications in the DFT+Σ procedure arise from the fact that we assume that the spiro molecules in the junctions are bonded to gold through thiolate bonds (see Fig. 3). Taking away the Au electrodes, the so-called "contacted molecule" in the junction thus forms a radical, since there are no hydrogen or acetyl groups attached to the three sulfur atoms of the tripod anymore. To determine HOMO and LUMO levels of a chemically inert molecule, we assume that the respective spiro molecule in the gas-phase features thiol SH endgroups at its legs instead (see Supplementary Fig. 8). We identify the HOMO and LUMO levels at the gas-phase molecule, and compute there $\epsilon_{\mathrm{H}}, \epsilon_{\mathrm{L}}$ as well as IP, EA. Image charge corrections $\Delta_{\mathrm{occ}}, \Delta_{\mathrm{virt}}$ are, however, computed at the contacted molecule, since the junction geometry enters. We have checked, as is visible in Supplementary Fig. 11, that in the contacted molecule there are molecular orbitals of nearly identical shape as the HOMO and LUMO states of the gas-phase one. $\Delta_{\mathrm{occ}} = -\Delta_{\mathrm{HOMO}}, \Delta_{\mathrm{virt}} = \Delta_{\mathrm{LUMO}}$ are thus computed from the charge densities of these corresponding HOMO and LUMO states of the contacted molecule. Finally, Σ_{occ} is applied to the contacted molecule as a shift of the gas-phase-related HOMO state and all states with energies below it, while Σ_{virt} is used for the gas-phase-related LUMO and all states with energies above it. The magnitude of all the different contributions entering into $\Sigma_{\mathrm{occ}}, \Sigma_{\mathrm{virt}}$ is listed in Supplementary Table 2.

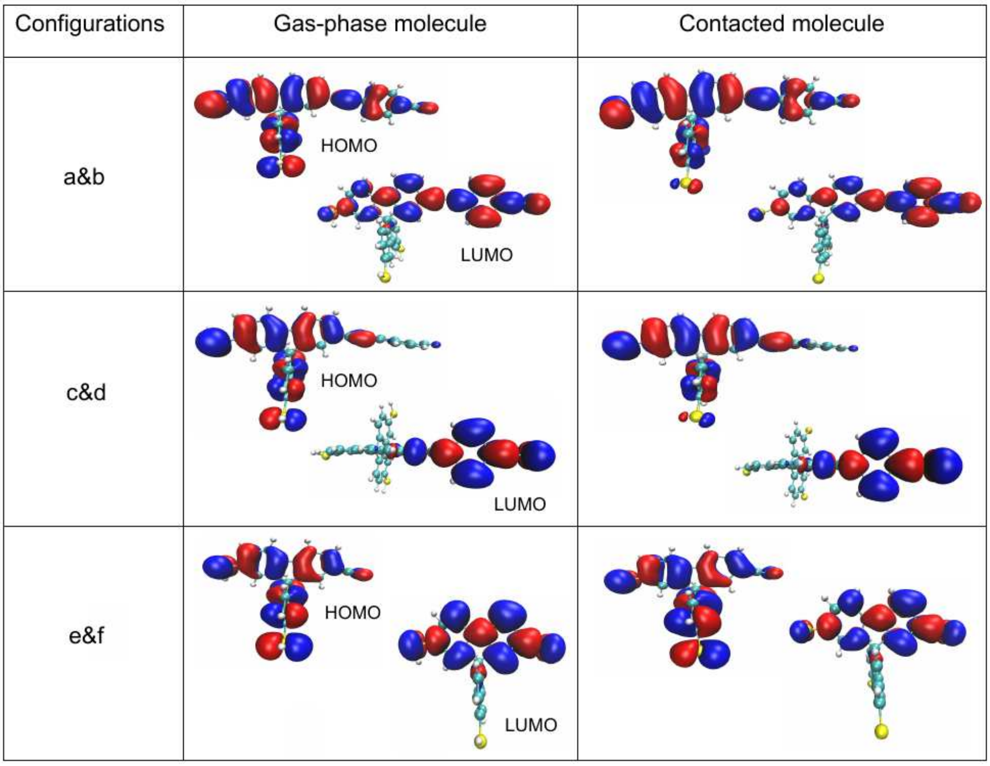

Supplementary Figure 11. HOMO and LUMO levels of the gas-phase molecules (with SH terminal groups at the tripodal feet) and the corresponding orbitals of the contacted molecules (with S terminations) for the molecular structures shown in Fig. 3a-f.

Supplementary Note 8. SIMULATED STM IMAGES

Based on the Tersoff-Hamann model [5], we calculated STM images of isolated spiro A, spiro A - rotated ring, and spiro B molecules (see Supplementary Fig. 8a-c). In this way, we can check the validity of our theoretical models and better interpret the experimental results. Within this approach, simulated STM images emerge from local densities of states (LDOS) near the Fermi energy. According to the positive bias voltage applied in the measurements, the unoccupied orbitals contribute. The wave functions that are needed for calculating these states (such as the LUMO) are obtained from DFT. In Supplementary Fig. 12 and Supplementary Fig. 13 the STM images based on the LUMO as well as the addition of LUMO and LUMO+1 are shown. The simulated constant height STM images confirm our adsorption model presented in Fig. 1, in particular the assumption that the phenyl ring in the head group of spiro A is rotated with respect to the gas-phase relaxation.

Configuration	$-\text{IP} - \epsilon_\text{H}$	$-\text{EA} - \epsilon_\text{L}$	Δ_occ	Δ_virt	Σ_occ	Σ_virt
a	-1.354	1.464	0.846	-0.539	-0.508	0.925
b	-1.392	1.552	0.839	-0.539	-0.553	1.013
c	-1.413	1.543	0.938	-0.556	-0.475	0.987
d	-1.475	1.623	0.927	-0.556	-0.548	1.067
e	-1.543	1.711	1.117	-0.755	-0.425	0.957
f	-1.542	1.705	1.337	-0.756	-0.205	0.949

Supplementary Table 2. Relevant energies in the DFT+Σ method for the spiro molecules on Au(111) in the different configurations shown in Fig. 3a-f. a-d, Spiro A, e,f, spiro B. All energies are given in units of eV.

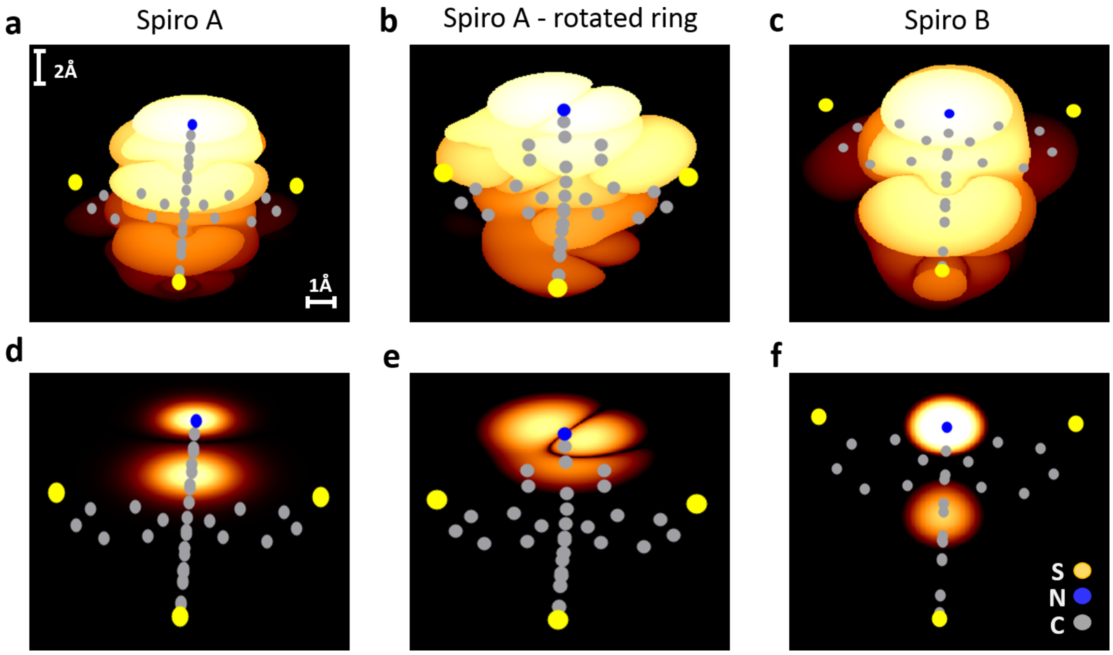

Supplementary Figure 12. Simulated STM images determined from the LDOS of the LUMO. a-c, Constant current mode, **d-f**, constant height mode for the molecules shown in Supplementary Fig. 8a-c.

Supplementary Note 9. ADSORPTION CONFIGURATION OF SPIRO B

The submolecular structure of spiro B, consisting of two lobes that can be seen in constant height images (see Fig. 1d), agrees well with the simulated STM images (see Supplementary Fig. 14a and Supplementary Fig. 12f). Comparison of the constant current image of spiro B

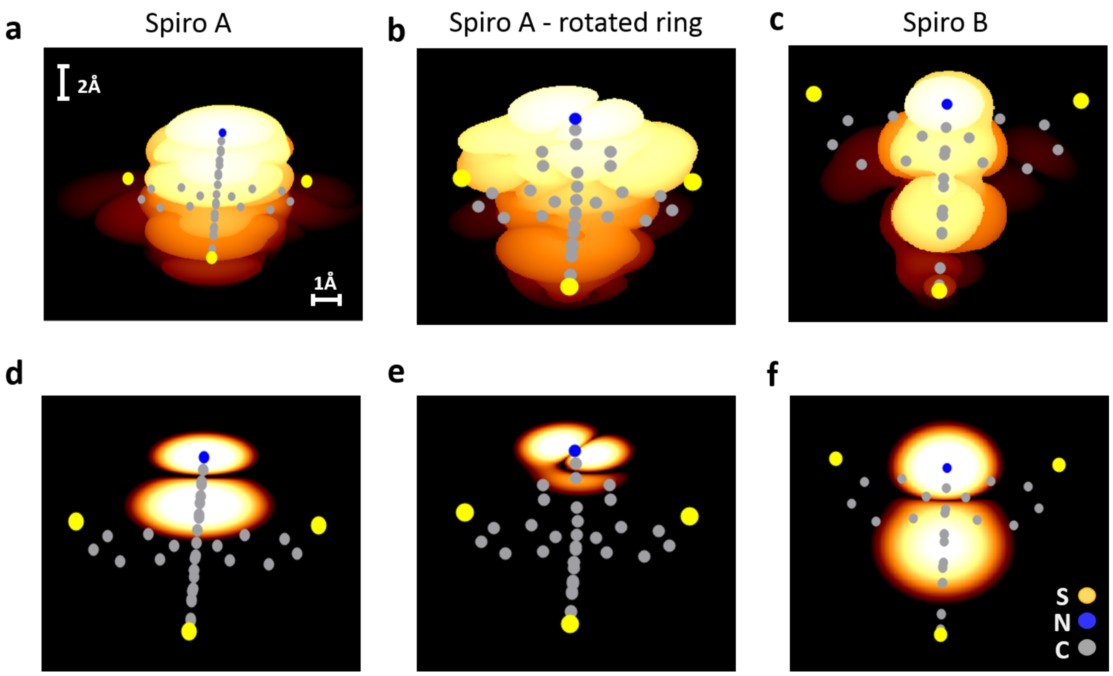

Supplementary Figure 13. Simulated STM images determined from the LDOS of both the LUMO and LUMO+1. a-c, Constant current mode and, **d-f**, constant height mode for the molecules shown in Supplementary Fig. 8a-c.

with the corresponding simulation further supports our proposed adsorption configuration (see Supplementary Fig. 14b). The ordered rows of spiro B (see Supplementary Fig. 14d) allow for a directional histogram that is presented in Supplementary Fig. 14c. There are only six preferred orientations of the molecular rows deviating by 5 ° from the densely packed directions of Au(111), indicating a well defined adsorption configuration. The STM image of spiro A clearly shows an asymmetric arrangement of two darker lobes at the position of the head group which can be identified as a signature of the rotated phenyl ring (see Supplementary Fig. 14e and Supplementary Fig. 13b,e).

Supplementary Note 10. MECHANICALLY INDUCED CONTACT FORMATION FOR SPIRO B

The hysteresis in the I-z curves presented in Fig. 2a,b depends on the lateral position of the tip with respect to the nitrile group as is revealed in the following. Supplementary Figure 15a-c shows the results of a map of I-z curves recorded at each pixel above the island

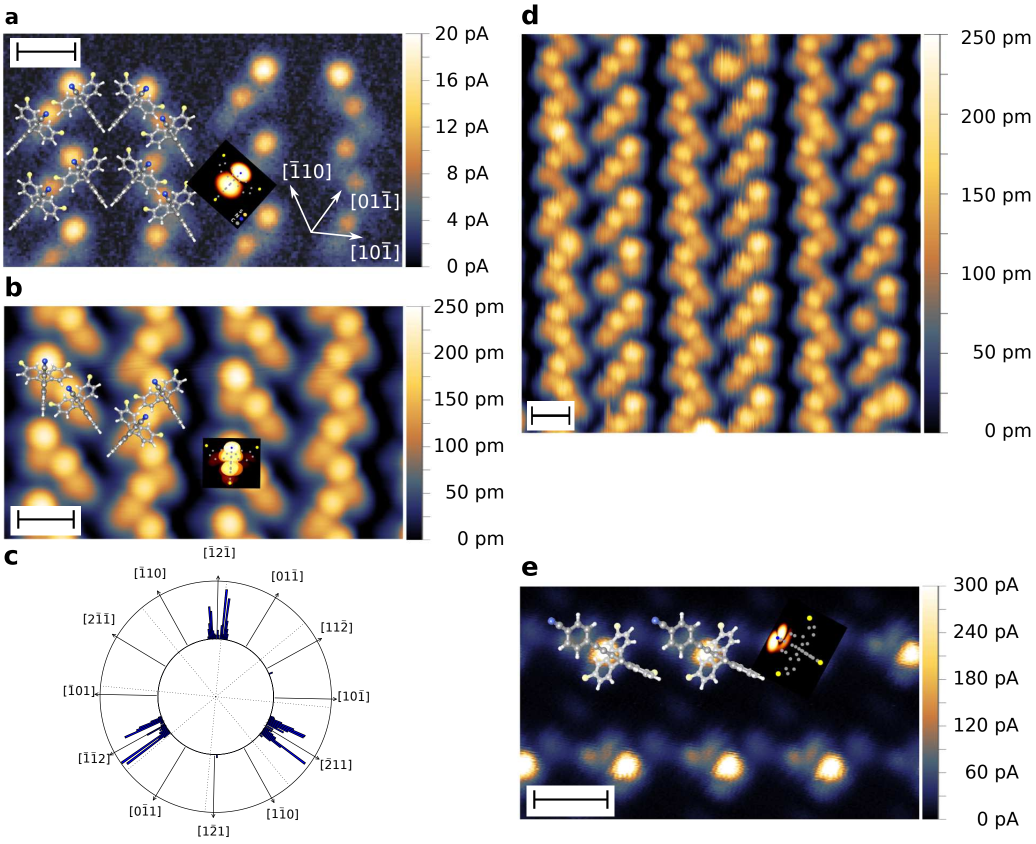

Supplementary Figure 14. **Orientation of the spiro molecules**. **a**, Constant height image of an ordered layer of spiro B (700 mV). The lattice directions of the substrate are indicated. **b**, Constant current image of an ordered layer of spiro B (1 V, 30 pA). **c**, Distribution of the orientations of braided rows of spiro B. **d**, 10×10 nm^2 view of ordered spiro B molecules. **e**, Constant height image of an ordered layer of spiro A without contact formation (2.6 V). In the panels **a**,**b**,**e** the insets show the corresponding simulated images, molecular models are superimposed to scale. Length of the scale bars is 1 nm.

of spiro B that is shown in Fig. 4a and in Supplementary Figure 14a. The feedback loop was not used in this measurement, and the *I*-*z* curves start at a plane of constant height above the surface. The critical distance z_{ON}, at which the contact is closed, is mapped in Supplementary Fig. 15a and reproduces the ellipsoidal areas of maximum apparent height of the molecule in the corresponding topographic image (see Fig. 1d and Supplementary Fig. 14a). Supplementary Figure 15b shows the distances z_{OFF} at which the contact re-opens upon retraction of the tip. This map also displays a mirror symmetry with respect to the backbone of the molecule, but unlike Supplementary Fig. 15a it shows a monotonous

variation along the axis of the backbone. This is also reflected in the map of the vertical hysteresis distance (see Supplementary Fig. 15c), that is, the difference between z_{OFF} and z_{ON}: Each molecule shows up as a characteristic c-shaped area of large hysteresis indicating a high binding energy between tip and molecule. This map of the hysteresis can be compared to simulations of the total energy which also reflect the asymmetry with respect to a displacement of the tip along the molecular backbone (see Supplementary Fig. 15d and Supplementary Figure 10 for details). At the rim of the ellipsoid, towards the center of the molecule (Supplementary Fig. 15e), the molecular junction largely stays closed upon retraction and the hysteresis is enhanced, while the bond breaks earlier and the hysteresis is reduced when the tip is placed laterally in front of the nitrile group (Supplementary Figure 15f). This behavior is also observed for the electric-field induced contacting presented in Supplementary Figure 18 and is in full agreement with the proposed adsorption model and further emphasizes the need for a precise lateral control over the bond configuration in order to produce reliable molecular junctions. Note that molecules of the same orientation on the surface behave identically, which excludes a plastic reorganization of the junction and indicates a well-defined and reproducible situation which allows for a more detailed analysis of the energetics of bond formation and elastic molecular deformation. This is in agreement with previous studies which have shown that the nitrile terminal group is well-suited for highly reproducible contact formation to Au electrodes [6]. In contrast to the detailed study of contact formation between a metallic tip and a single CO molecule by Welker and Giessibl [7] and the experiments on forces during atomic manipulation [8], the gold-nitrile bond discussed here does not show a simple rotational symmetry of the energies involved in bond formation and molecular deformation but reproduces the reduced, twofold symmetry of the tilted molecular head group. Slight deviations from this characteristic shape seen on molecules of different orientation are probably caused by a non-symmetric arrangement of atoms at the tip apex.

Supplementary Note 11. POSITION DEPENDENCE OF THE ELECTRICALLY INDUCED CONTACT FORMATION

As discussed in the main text, voltage-induced switching is only possible when the elastic energy needed to stretch the molecule is comparable to the energy of the tip-molecule bond.

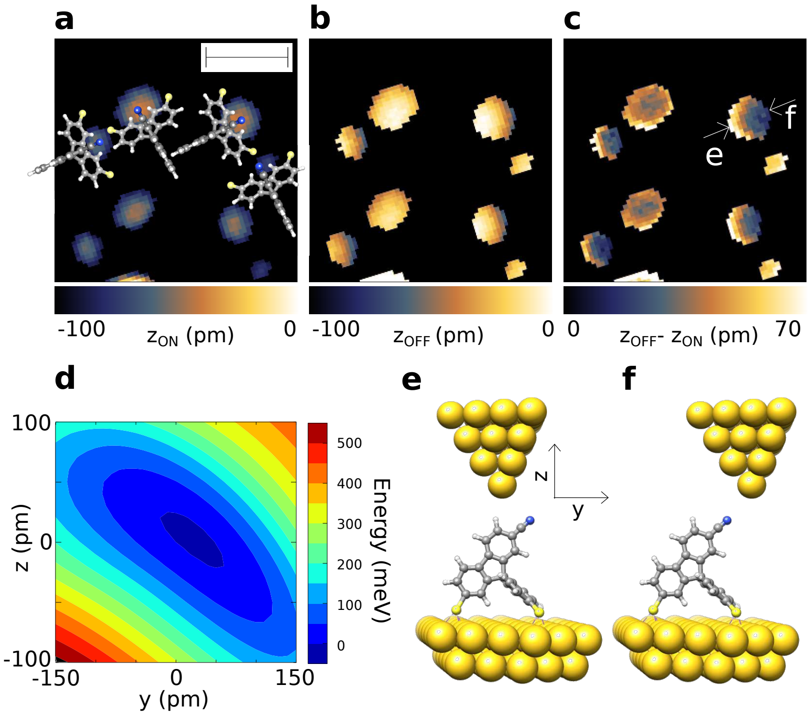

Supplementary Figure 15. Contact formation by approaching the tip towards the sample. a-c, Maps constructed from *I*-*z* curves recorded at every pixel on spiro B molecules at a bias of 70 mV: **a**, critical position z_{ON} for closing the contact (length of the scale bar is 1 nm, molecular structure superimposed to scale), **b**, critical position z_{OFF} for opening the contact, **c**, hysteresis in z. **d**, Calculated total energy of the molecular junction as a function of distance between tip and sample (z-direction) and displacement of the tip along the molecular backbone (y-direction). Configuration of the molecular junction at the tip positions indicated in **c**: large hysteresis is observed for the configuration **e**, weak hysteresis is observed for the configuration **f**.

Therefore the threshold voltages that lead to switching strongly depend on the distance between tip and sample. *I*-*V*-curves were taken at six different tip-sample distances above the very same spiro A molecule. Supplementary Figure 16a-c shows measurements at three representative distances. Note that the absolute position is unknown while the relative position is precisely defined by the voltage applied to the piezo tube. Clearly, the threshold voltages shift towards lower values with increasing tip-sample distance. All threshold voltages are plotted in Supplementary Fig. 16d. The observed behavior can be understood as an increase in elastic energy which has to be compensated by the dipole energy in the electric field. At a tip position of 1.05 nm almost no electric field is necessary to induce contact

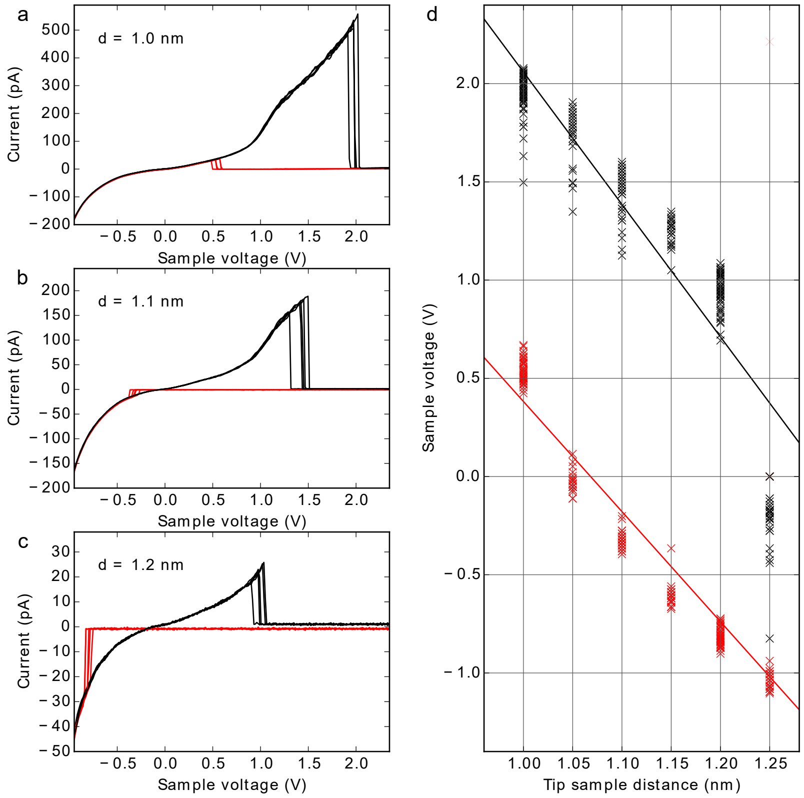

Supplementary Figure 16. Threshold voltage for switching at different heights d above spiro A. a-c I-V curves at 1 nm, at 1.1 nm, and at 1.2 nm, respectively. **d**, Threshold voltages as a function of tip-sample distance. The fitted straight line is a guide to the eye.

formation, because the elastic energy exactly balances the bond energy. At a distance of 1.0 nm, the tip is so close that contact formation happens even at a positive voltage which effectively repels the molecular head group. These observations rule out mechanisms related to inelastic excitations or tunneling to specific orbitals.

Similar to the mechanical contacting experiments presented in Fig. 4 and Supplementary Fig. 15, the hysteresis for electric-field induced contact formation delicately depends on the position of the tip with respect to the head group of the molecule. Supplementary Fig. 17a-c shows maps of the voltage-controlled switching behavior of spiro B, where I-V curves similar to those in Fig. 5a,b were recorded and analyzed at each pixel. The maps presented in Supplementary Fig. 17a-c were recorded at constant height above the substrate, and the voltage was swept at each pixel between -1 V and 2 V and back. The critical voltage that is needed to close the contact between tip and molecule (see Supplementary Fig. 17a) again reproduces the symmetric ellipsoidal shape of the molecular head group. In the center,

even at a positive voltage (which effectively repels the molecular head group), the molecule closes the contact. We interpret this as a configuration at which tip and molecular head group are positioned at similar heights above the substrate. The higher sample voltage that is necessary to open the contact again shows a characteristic c-shape of lower symmetry than the voltage needed to close the contact (see Supplementary Fig. 17b). Similar to Supplementary Fig. 15c, a pronounced hysteresis is observed when the tip is positioned closer to the molecular center, while very low hysteresis is found when the tip is laterally positioned farther away from the center of the molecule (see Supplementary Fig. 17c and Supplementary Fig. 15e,f). The spatial distribution of the critical voltages for closing and opening the contact is clearly dictated by the orientation of the molecules so that an effect related to the shape of the tip apex [7] can be safely excluded. These observations of the spatial variation of the electrostatic contact formation are in full agreement with the experiments and simulations on the mechanical contact formation presented in Figs. 2,4. As can be seen in Supplementary Fig. 17e, the threshold voltage varies continuously with the position relative to the molecule. Finally, the conductance of the junction was derived from the I-V curves and is shown in Supplementary Fig. 17d. Again, each molecule shows up as a characteristic shape with a variation of the conductance along the axis of the molecular backbone. However, this variation is small compared to the difference between ON and OFF state conductances and, similar to the mechanically induced switching presented in Figs. 2,4, the corresponding conductance histograms shown in Supplementary Fig. 17f,g demonstrate that the ON and the OFF states can be identified by clearly separated conductance values.

Supplementary Note 12. ENERGY DIFFERENCES BETWEEN THE OPEN AND CLOSED MOLECULAR CONTACT FOR SPIRO B

In analogy to the measurements on spiro A presented in the manuscript, the molecular junction formed with spiro B can be driven into a thermally bistable state by spatially positioning the tip and choosing the electric field appropriately. Again, we measured the energy difference between the two metastable states as a function of the applied bias voltage, that is, as a function of the applied electric field. Supplementary Figure 18a shows the time trace of the thermally activated statistical switching of the conductance at a bias voltage of about 240 mV. The population of the ON and the OFF state depends on the bias voltage.

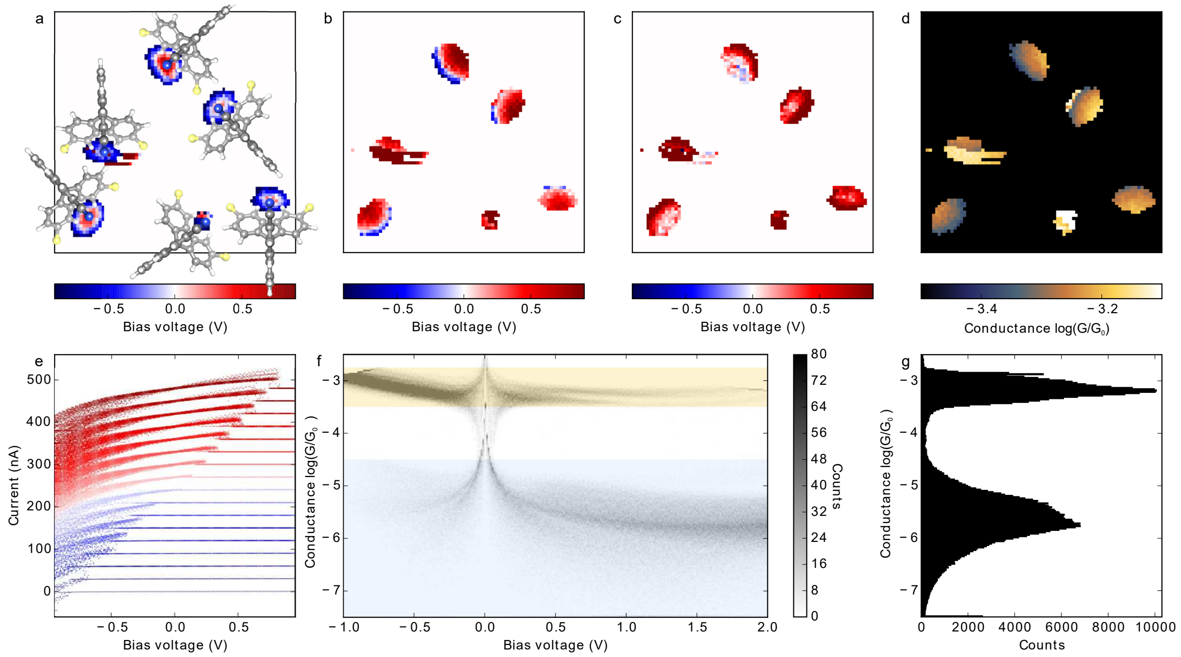

Supplementary Figure 17. **Contact formation to spiro B by electric fields**. **a-c**, Maps constructed from *I*-*V* curves recorded at constant height at every pixel: critical voltage for closing the molecular contact (sweep from positive to negative voltage) with molecular structure superimposed to scale (**a**), critical voltage for opening the molecular contact (sweep from negative to positive voltage, **b**), voltage hysteresis (**c**). **d**, Map of the conductance of the electrically closed junction at -0.3 V. **e**, *I*-*V* curves for opening the junction with positions color coded according to panel **b**. An offset is added to each curve for clarity. **f,g**, Corresponding conductance histograms constructed from all *I*-*V* curves that lead to contact formation. The diverging conductance at low voltages, that is at low currents, is an artifact related to imperfect offset correction.

Supplementary Fig. 18c shows the time trace of the current as function of the applied voltage which was slowly swept from 0 mV to 400 mV. In addition to the voltage window around 240 mV shown in Supplementary Fig. 18a, two enlarged views are shown for 220 mV and 300 mV. The voltage-dependent population of the ON state ($N_{\mathrm{ON}}/(N_{\mathrm{OFF}} + N_{\mathrm{ON}})$), indicated by red crosses, is then translated into the energy difference between the ON and the OFF state (black crosses in the lower panel of Supplementary Figure 18c) according to equation (2) of the main manuscript. Again, this energy difference scales linearly with the applied voltage and indicates an electric-field controlled tilt of the dipole in full agreement with the experiments on spiro A. Furthermore, the slope of $a = -9.7\,\mathrm{meV\ V}^{-1}$ allows to

extract quantitative information about the tilt angle of the dipole moment. To this end, we calculated the dipole moments of the two molecules to $|\mathbf{p}| = 120\,e\text{pm}$ for spiro A and $|\mathbf{p}| = 105\,e\text{pm}$ for spiro B (see table S2). With this, the slope is given by

$$a = \frac{\partial(E_{\text{OFF}} - E_{\text{ON}})}{\partial V} = \frac{|\mathbf{p}|}{z_{\text{tip}}} \cdot (\cos \Phi_{\text{OFF}} - \cos \Phi_{\text{ON}}). \tag{1}$$

Furthermore, we estimated the distance z_{tip} between tip and sample surface from the tunneling current and the bias voltage. In the contact regime, the tip position can be identified with the height of the molecule. This value amounts to about 1100 pm in the case of spiro A and is slightly lower than the height of the molecule in the gas phase relaxation (1340 pm) which exhibits an angle of $\Phi_2 = 52°$ (see Supplementary Fig. 8). On spiro B the height in contact was estimated to 950 pm. Based on these values, we estimated the change of the tilt angle of the dipole moment to about 16° when switching between the ON ($\Phi_{\text{ON}} = 52°$) and OFF states ($\Phi_{\text{OFF}} = 68°$) in the case of spiro A. In the case of spiro B, the change in tilt of the dipole moment amounts to about 7°.

In analogy to the experiment presented in Fig. 6e, we measured the energy difference between the open and closed molecular junction as a function of the distance between tip and sample for spiro B. Supplementary Fig. 18b shows the corresponding time trace of the current for a distance variation of 20 pm for spiro B at constant voltage. Again, we observe a continuous transition of the population of the ON state to the OFF state with increasing distance between tip and sample. The slope of the fitted straight line is $-0.26\,\text{meV pm}^{-1}$ which, in other units, corresponds to a force of 42 pN.

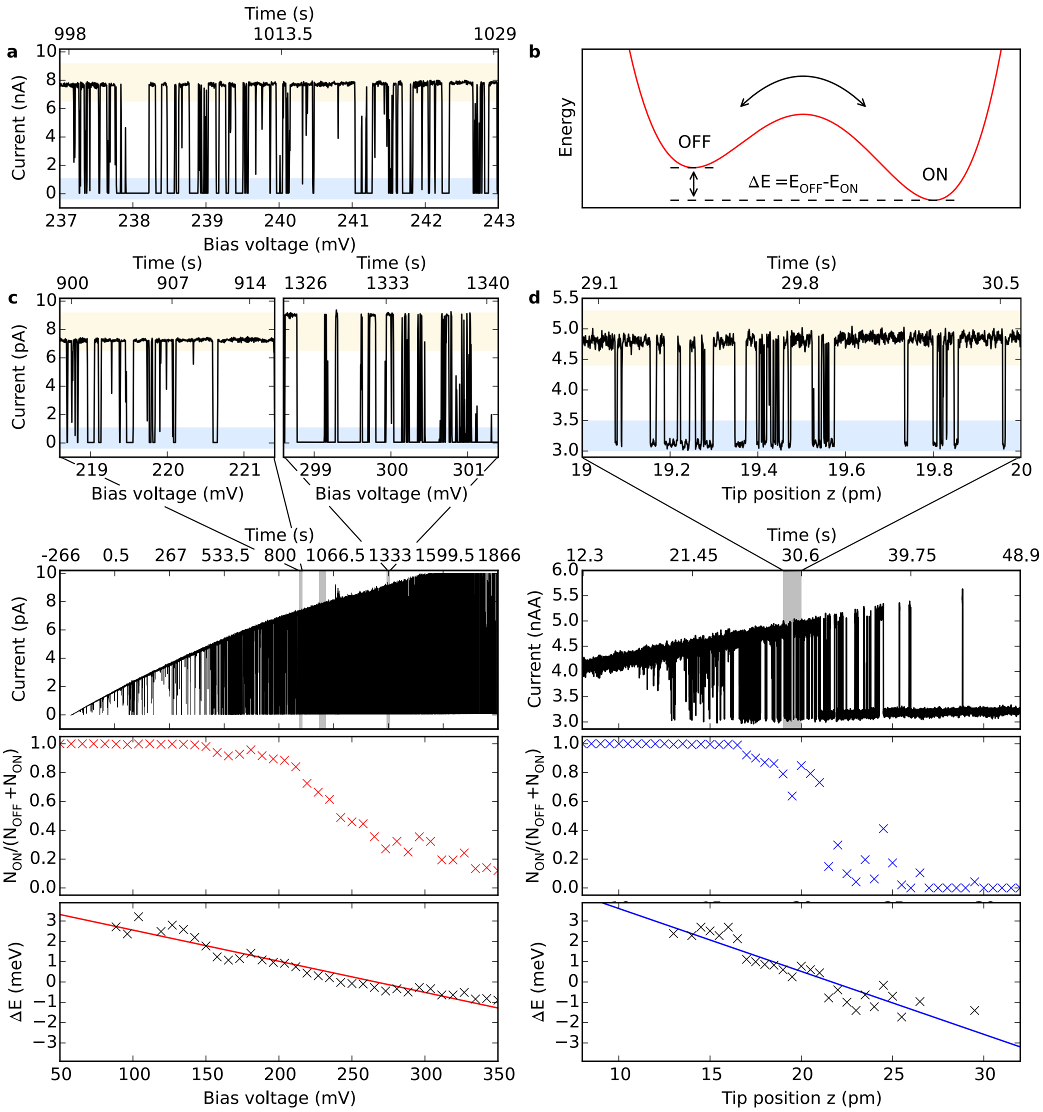

Supplementary Figure 18. Energy differences between the open and closed molecular contact for spiro B. a, Statistical conductance switching of spiro B at a bias voltage of 240 mV. **b**, Model of a double-well potential for ON and OFF states of the molecular head group. **c**, Statistical switching as a function of applied bias voltage and time. In addition to the enlarged view shown in **a**, two voltage intervals marked in grey are shown in enlarged views (220 mV and 300 mV). The corresponding population of the ON state $N_{\mathrm{ON}}/(N_{\mathrm{OFF}} + N_{\mathrm{ON}})$ is indicated by red crosses, the resulting energy difference between ON and OFF states derived from Boltzmann statistics is indicated by black crosses, the red line is a linear fit to the data. **e**, Statistic switching recorded at 500 mV as a function of time and distance between tip and sample, corresponding population of the ON state (blue crosses) and resulting energy difference between ON and OFF states (black crosses, linear fit in blue).

Supplementary References

---

[1] Valášek, M. *et al.* Synthesis of Molecular Tripods Based on a Rigid 9,9'-Spirobifluorene Scaffold. *J. Org. Chem.* **79**, 7342–7357 (2014).

[2] Quek, S. Y. *et al.* Amine-gold linked single-molecule circuits: experiment and theory. *Nano Lett.* **7**, 3477–3482 (2007).

[3] Mowbray, D. J., Jones, G. & Thygesen, K. S. Influence of functional groups on charge transport in molecular junctions. *J. Chem. Phys.* **128**, 111103 (2008).

[4] Zotti, L. A. *et al.* Heat dissipation and its relation to thermopower in single-molecule junctions. *New J. Phys.* **16**, 015004 (2014).

[5] Tersoff, J. & Hamann, D. R. Theory and application for the scanning tunneling microscope. *Phys. Rev. Lett.* **50**, 1998–2001 (1983).

[6] Mishchenko, A. *et al.* Single-Molecule Junctions Based on Nitrile-Terminated Biphenyls: A Promising New Anchoring Group. *J. Am. Chem. Soc.* **133**, 184–187 (2011).

[7] Welker, J. & Giessibl, F. J. Revealing the Angular Symmetry of Chemical Bonds by Atomic Force Microscopy. *Science* **336**, 444–449 (2012).

[8] Ternes, M., Lutz, C. P., Hirjibehedin, C. F., Giessibl, F. J. & Heinrich, A. J. The Force Needed to Move an Atom on a Surface. *Science* **319**, 1066–1069 (2008).
